# Reference Genome Anchoring of High-Density Markers for Association Mapping and Genomic Prediction in European Winter Wheat

**DOI:** 10.3389/fpls.2019.01278

**Published:** 2019-11-08

**Authors:** Olufunmilayo Ladejobi, Ian J. Mackay, Jesse Poland, Sebastien Praud, Julian M. Hibberd, Alison R. Bentley

**Affiliations:** ^1^The John Bingham Laboratory, NIAB, Cambridge, United Kingdom; ^2^Department of Plant Sciences, The University of Cambridge, Cambridge, United Kingdom; ^3^IMplant Consultancy Ltd., Chelmsford, United Kingdom; ^4^Wheat Genetics Resource Center, Department of Plant Pathology, Kansas State University, Manhattan, KS, United States; ^5^Biogemma, Site de La Garenne, Chappes, France

**Keywords:** mapping, quantitative traits, trait dissection, next-generation sequencing, genomic selection

## Abstract

In this study, we anchored genotyping-by-sequencing data to the International Wheat Genome Sequencing Consortium Reference Sequence v1.0 assembly to generate over 40,000 high quality single nucleotide polymorphism markers on a panel of 376 elite European winter wheat varieties released between 1946 and 2007. We compared association mapping and genomic prediction accuracy for a range of productivity traits with previous results based on lower density dominant DArT markers. The results demonstrate that the availability of RefSeq v1.0 supports higher precision trait mapping and provides the density of markers required to obtain accurate predictions of traits controlled by multiple small effect loci, including grain yield.

## Introduction

Historically, wheat breeding has focused on phenotypic selection for final yield potential combined with morphological and disease resistance traits (Cavanagh et al., 2013). The advent of genetic and genomic tools has largely supported marker-assisted selection for major genes in segregating generations. There is additional potential for the introgression of favorable genetic regions controlling variation in agronomically significant quantitative trait loci (QTL) through the routine application of genomic selection (GS) schemes that are based on the combined merit of genome-wide markers (Meuwissen et al., 2001; Stamp and Visser, 2012).

Advances in genomic technologies combined with computationally efficient statistical models present new opportunities for molecular crop breeding. Selection based on phenotype is complex, time-consuming, and still costly; thereby necessitating the adoption of molecular breeding systems. For crop geneticists and plant breeders, the adoption and applicability of genotyping-by-sequencing (GBS) has been recently demonstrated for a wide range of crops. This includes the detection of QTL controlling agronomic traits in rice and soybean (Begum et al., 2015; Sonah et al., 2015) and the detection of introgressions in cotton, *Brassica*, and sorghum (Kim et al., 2016).

GBS is an attractive alternative to array-based methods for generating high volume genome-wide single nucleotide polymorphisms (SNPs) for genome-wide association studies (GWAS) and GS. It is a fast, robust, and high-throughput method applicable across species in which genotyping and polymorphism discovery occur simultaneously, thereby avoiding the upfront effort of discovering, screening, and characterizing polymorphisms that is generating such ascertainment bias (Poland and Rife, 2012). Initially developed by Elshire et al. (2011), the technique was modified by Poland et al. (2012b) to produce a two enzyme version suitable for polyploid species with large genomes. This uses a combination of methylation sensitive restriction enzymes, *PstI* and *MspI*, cutting at rare and common restriction sites, respectively followed by next-generation sequencing. An accompanying bioinformatics pipeline, Tassel-GBS (Glaubitz et al., 2014), is in place for calling SNP variants from the resulting GBS sequences. GBS has recently been employed in wheat for linkage mapping and genomic prediction studies (Poland et al., 2012a; Poland et al., 2012b; He et al., 2014) and the availability of a high-quality reference RefSeq v1.0 genome assembly (International Wheat Genome Sequencing Consortium, 2018) should enhance the efficiency and quality of GBS data for downstream analysis (Kim et al., 2016).

GWAS combines high-density genome wide marker information (such as those derived from GBS) with high levels of genetic diversity in panels of individuals in order to map QTL. In a breeding context, it is used to detect genomic regions controlling complex quantitative traits and identifying alleles (and associated markers) for exploitation in variety improvement. GWAS has been used to detect marker-trait associations for several traits in wheat including grain protein content, thousand kernel weight and specific weight (Reif et al., 2011), agronomic traits (Bentley et al., 2014; Mora et al., 2015), and resistance to Fusarium Head Blight (Arruda et al., 2016).

Despite the power of GWAS to detect significant associations, many agronomically important traits under selection are polygenic, meaning these traits are influenced by many common SNPs, each with small individual effect, and remain recalcitrant to conventional marker-assisted selection. GS was proposed to address this complexity (Meuwissen et al., 2001) because it omits the significance testing used in GWAS, modelling the effect of all genotyped markers simultaneously (Meuwissen et al., 2001). This avoids the “Winners’ Curse” bias (Beavis, 1994) caused by selection of a subset of markers, and also improves the accuracy of selection. By including genome-wide marker data in a model to predict complex traits, the accuracy of selection is increased through greater capture of low heritability traits. This could accelerate genetic gain through a shortening of the breeding cycle, particularly for traits that are expensive to phenotype, are measured late in the growing season or require large volumes of seed to assess.

Several studies have investigated the accuracy of prediction using real and simulated data. The central considerations in these studies have been the predictive ability of available statistical models and the composition and size of the training population (Heslot et al., 2012; Daetwyler et al., 2013; de los Campos et al., 2013). Using eight datasets from four plant species including wheat and barley, Heslot et al. (2012) tested 11 GS models and found predictive abilities to be equivalent for many of the methods but with differences in computational times. Ridge regression best linear unbiased prediction (RR-BLUP), is computationally efficient (Endelman and Jannink, 2012; Lipka et al., 2015) and is used in the present study to assess predictive variation between genetic marker sets.

In this study, a previously described panel of 376 elite winter wheat varieties released or commercialized in the UK, France, and Germany between 1946 and 2007 (Bentley et al., 2014) were genotyped with GBS to provide dense genome-wide marker coverage. By re-genotyping the panel, we aimed to compare GWAS and GS performance across low- and high-marker density genotyping platforms and demonstrate the use and applicability of GBS given the recent release of a high-quality International Wheat Genome Sequencing Consortium (IWGSC) RefSeq v1.0 genome assembly (International Wheat Genome Sequencing Consortium, 2018). We tested GBS as an effective means to identify large numbers of SNPs to detect broadly relevant QTL controlling key traits with high precision and to demonstrate the usefulness of GBS for GS and its potential for breeding applications.

## Materials and Methods

### Plant Material and Phenotyping

The previously described TriticeaeGenome panel consisting of 376 elite winter wheat varieties was used in this study (Bentley et al., 2014). The panel was evaluated for a range of agronomic traits in replicated European trials in France (FRA), Germany (DEU), and the UK (GBR) in 2010 and 2011 as described in Bentley et al. (2014). Flowering time (FT), grain yield (GY), and plant height (PH) were evaluated across all trials while nine additional traits including presence/absence of awns (Awns), winter kill (Wkill), maturity (MAT), grain protein content (Gpt), ears/m^2^ (Ears), lodging resistance (LR), grain specific weight (GSW), tiller number (TN), and thousand grain weight (TGW) were scored in single European locations as described in Bentley et al. (2014). All trait data are summarized in **Supplementary Table S1** and available from Figshare DOI: 10.6084/m9.figshare.7350284. For each trait, best linear unbiased estimates (BLUEs) were generated in GenStat (Payne, 2009) for variety performance at each site and over all sites for use in association analysis and genomic prediction. Marker-trait association for FT and GY was calculated on BLUEs for each site per year and overall values from all sites. Association for PH and LR was calculated from overall site BLUEs.

### Genotyping, Variant SNP Calling, and Imputation

Genomic DNA was isolated from 2-week-old seedlings of each line using a modified Tanksley extraction protocol (Fulton et al., 1995). GBS was conducted as described by Poland et al. (2012b). To ensure adequate sequencing coverage and enhance accuracy each line was replicated four times with each replicate identified by a unique barcode. GBS libraries were sequenced in 96-plex across four flow cell lanes in Illumina HiSeq. Fastq sequence files were processed in the TASSEL GBS pipeline version 5.2.31 (Glaubitz et al., 2014). Reads were trimmed to 64 base pairs and filtered based on sequence quality score to obtain only good quality reads with a barcode sequence, five nucleotides of *PstI* restriction site fragment and no unreadable bases (N) in between. Reads were aligned to the IWGSC RefSeq v1.0 reference genome (International Wheat Genome Sequencing Consortium, 2018) using Bowtie2 (Langmead and Salzberg, 2012). SNP sites were filtered to remove loci with extremely low coverage or high levels of missing data and heterozygosity. Filtering also removed SNPs with minor allele frequencies (MAFs) below 5%. Individuals with more than 50% data missing were excluded from downstream analysis. The filtered GBS data in Hapmap format is available from Figshare DOI: 10.6084/m9.figshare.7350284. Missing SNPs were imputed using the LD-kNNi method implemented in TASSEL (Money et al., 2015) with the following parameters: number of sites in LD = 200; maximum number of nearest neighbors used in imputation = 50; 10 imputation iterations.

### Linkage Disequilibrium

Linkage disequilibrium (LD) was evaluated for average decay for each genome and between pairs of SNPs per chromosome. For the overall genome LD decay pattern, only SNP sites with MAFs of at least 0.1 were included. Pairwise LD was calculated as the squared correlation of allele frequency *r²* between SNP loci (Weir, 1996). The P-values of LD between any two loci were determined by a two-sided Fisher’s exact test. To summarize the pattern of decay of LD with distance, a curve of decay of *r²* with distance in base pairs was estimated by nonlinear least squares (Remington et al., 2001; Marroni et al., 2011). LD was estimated in TASSEL (Glaubitz et al., 2014). LD decay plots of *r²* values with distance in base pairs were plotted and the LD decay curve fitted in R (R Core Team, 2016).

### Population Structure

A subset of 7,865 uncorrelated SNPs derived from thinning the full set of SNPs based on physical distance (minimum distance 100,000 bp) was used for evaluation of population structure. Principal coordinate analysis (PCoA) was conducted in the R package “ade4” (Dray and Dufour, 2007). The first two principal coordinates accounting for the largest proportion of variation were used to visualize patterns of population structure within the panel. Population structure was also evaluated using the Bayesian clustering approach implemented in the software STRUCTURE 2.3.4 (Pritchard et al., 2000). A burn-in of 100,000 iterations followed by a Markov Chain Monte Carlo (MCMC) of 100,000 iterations was executed to estimate the number of subpopulations. An admixture model was applied for two to ten putative populations (K) and six independent runs were conducted for each K. The optimal K value was inferred based on the rate of change in log probability of data between successive K values using the *ad hoc* statistic, DeltaK (Evanno et al., 2005). The program CLUMPP was used to assign results from separate STRUCTURE runs to common populations (Jakobsson and Rosenberg, 2007). The panel had been previously genotyped with 2,012 polymorphic dominant Diversity Array Technology (DArT; www.diversityarrays.com) array markers and 1,804 markers retained for analyses. In this study, a subset of 1,117 unlinked DArT markers were reselected and used to re-estimate PCoA based on DArT.

### Association Mapping

Association was estimated by mixed linear modeling (MLM) implemented using the efficient mixed model association method (EMMA; Kang et al., 2008) in the Genomic Association and Prediction Integrated Tool (GAPIT; Lipka et al., 2012). To improve statistical power and exclude bias due to relatedness, the PCA + K mixed model (Yu et al., 2006; Zhang et al., 2010) was used. Within this model, population structure and relatedness were accounted for by jointly incorporating PCA as fixed effects and a kinship matrix as a random effect, respectively. The kinship matrix was estimated by the centered identity-by-state method derived by Endelman and Jannink (2012) in TASSEL. Bayesian information criterion (BIC) was used to determine the optimal number of principal components in the mixed model for estimating marker-trait association. A Bonferroni correction threshold for multiple testing was calculated at an experimental P-value = 0.01. The amount of phenotypic variation controlled by identified QTL was estimated as the difference in residual variance between models with and without the marker effect. Significance of associations was tested using a false discovery rate (FDR) P-value at a cutoff of 0.05 according to Benjamini and Hochberg (1995). Marker-trait association for FT and GY was calculated on BLUEs for each site per year and overall values from all sites. Association for PH and LR was calculated from overall site BLUEs. Association mapping results from the present study using GBS markers were compared to previous results on the panel using DArT markers (Bentley et al., 2014).

### Genomic Prediction

RR-BLUP as implemented in the R package “rrBLUP” (Endelman, 2011) was used to predict genomic estimated breeding values (GEBVs). The predictive ability of GBS and DArT markers were compared across the panel for all 12 traits using tenfold cross-validation. The panel was also split by country of origin (FRA, DEU, and GBR) and prediction accuracy assessed within each group by tenfold cross-validation. Training populations were assembled separately from FRA, DEU, and GBR with 192, 82, and 70 varieties, respectively and with each used to predict the phenotypes of varieties from the two remaining countries combined and separately. In all cases prediction accuracy was evaluated as the average Pearson’s correlation between the predicted GEBVs and the true phenotype value across 10 runs.

## Results

### Genotyping

Approximately 1.4 million good quality reads (defined as bar-coded reads of 64 nucleotides in length with high quality scores) were generated from alignment to IWGSC RefSeq v1.0 (International Wheat Genome Sequencing Consortium, 2018). There was an overall alignment rate of 91.28% and of these 20.54% aligned to unique positions and a total of 200,712 SNP sites were identified from the alignment. Sequencing coverage per allele per line was variable among all lines dependent on the quality of genomic DNA. However, because the lines were sequenced in replicates, the effect of low coverage was minimal. Filtering on low coverage eliminated 28% of the data. The data were further filtered to remove lines with >30% SNPs missing, and SNP sites with >20% data missing. Highly heterozygous SNP sites were also filtered to avoid confounding effects from homoeologous SNPs. After filtering, a total of 42,795 SNPs and 350 individuals were retained for subsequent analyses. The proportion of SNP markers across the three wheat genomes was highest on the B genome (52%) followed by the A genome (32%) and the D genome (10%), which was lowest, as expected. Chromosome 1B had the highest number of SNPs (4,878) while 4D had the least (240). Unmapped SNPs comprised 2% of the SNP dataset (**Supplementary Table S2**). MAF were slightly skewed in favor of lower values. MAFs for 22.5% of SNPs were within the range 5%–10% (**Supplementary Figure S1**).

### Linkage Disequilibrium

**Figure 1:**
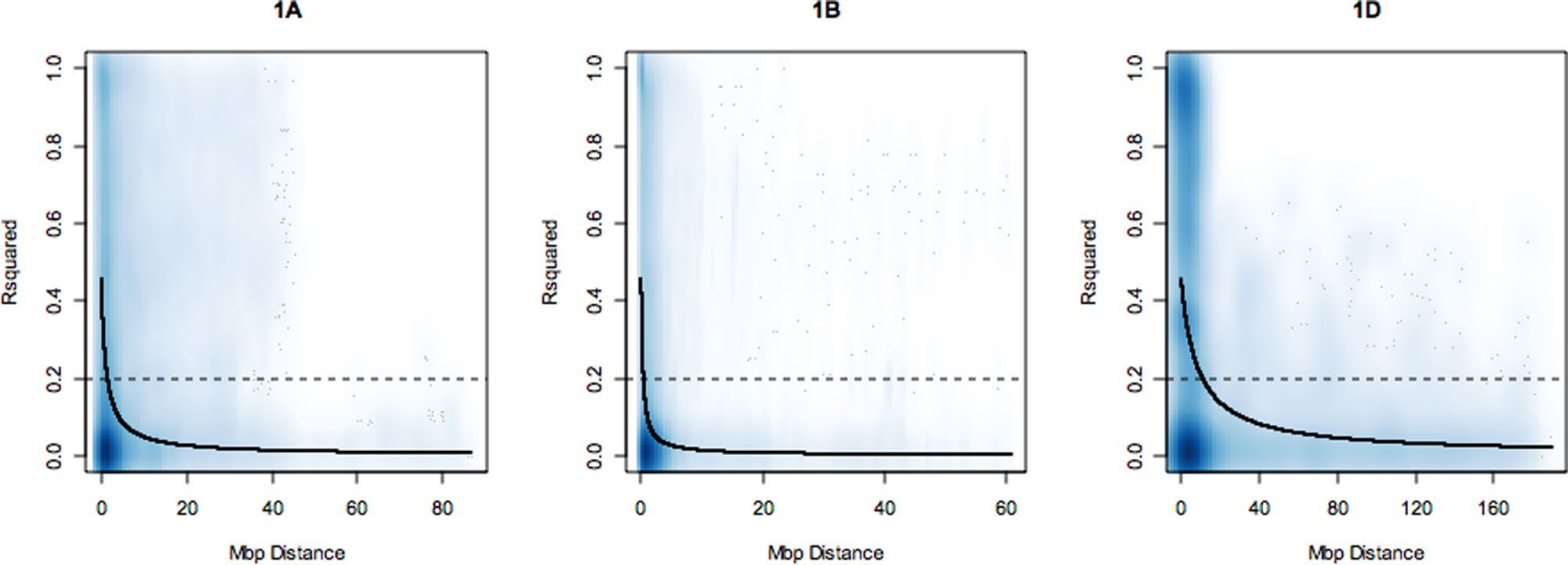
Linkage disequilibrium (LD) decay plots of r² over physical distance in mega base pairs (Mbp) on chromosomes 1A, 1B, and 1D. The dark blue line is the LD decay curve fitted by nonlinear least squares. LD decayed to r² of 0.2 (dotted line) at an approximate distance of 2.0, 1.0, and 10.0 Mbp on each group 1 chromosome, respectively. The deeper shades of blue in the graphs correspond to regions of the genome with high SNP marker density and lighter shades of blue corresponds to regions where SNP markers are less dense.

LD was estimated between SNP loci on each chromosome as the squared correlation of allele frequency *r²*. A nonlinear least squares curve was fitted to estimate the distance in mega base pairs (Mbp) within which LD decayed to 0.2 on each chromosome (summarized in **Supplementary Table S2**). Overall, LD decayed with increasing physical map distance on all chromosomes and on all genomes. However, on all chromosomes, some marker pairs separated by long distances were observed to be in high LD (r² = 1). Over the whole genome, LD decayed at an average distance of 4.98 Mbp. The slowest rate of LD decay was observed for the D genome followed by the B and A genomes with average LD decay distance estimates of 6.4, 4.5, and 4 Mbp, respectively. The average trend of LD decay rate estimated across each genome revealed that the percentage of SNP loci pairs with *r²* values above 0.2 on the A, B, and D genomes were 28.61%, 25.55%, and 19.37%, respectively. On the D genome, LD decay distance ranged from 2.5 Mbp (4D, 7D) to 10 Mbp (1D, 2D, 3D). On the B genome, the highest LD was observed on chromosome 2B at 10 Mbp and the lowest on 1B (1.0 Mbp). On the A genome, LD decay distance was 5 Mbp on chromosomes 4A, 5A, 6A, 7A. LD decay plots for 1A, 1B, and 1D are shown in **Figure 1**. LD decay plots for all other chromosomes are shown in **Supplementary Figure S2**.

### Population Structure

**Figure 2:**
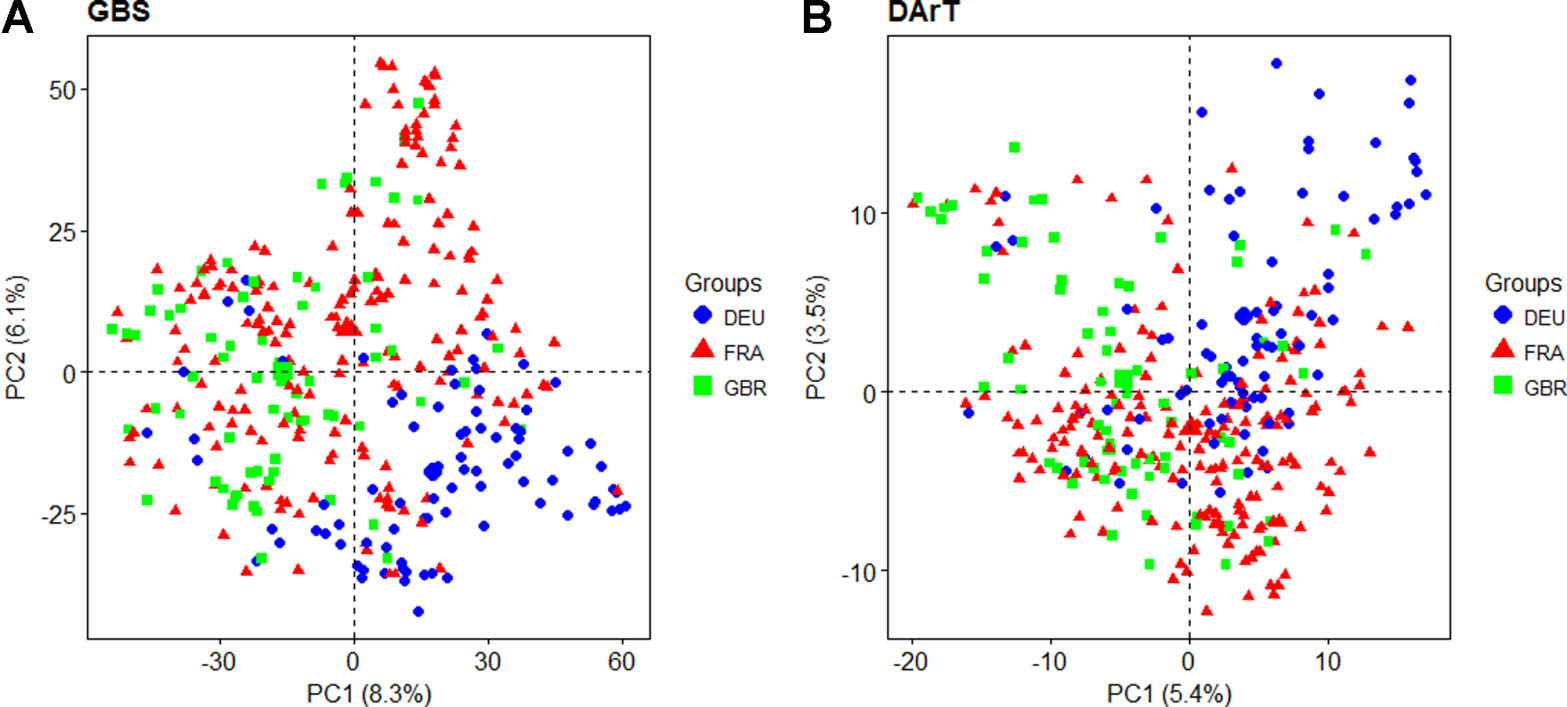
Principal coordinate analysis based on the first two principal coordinates using **(A)** Genotyping-by-sequencing (GBS) markers and **(B)** Diversity Array Technology (DArT) markers. Each point represents a line in the variety collection colored by its country of origin (DEU: blue; FRA: red; GBR: green).

Principal coordinate analysis was used to estimate and visualize population structure within the panel based on a subset of 7,865 evenly distributed GBS markers compared to the 1,117 dominant DArT markers that were previously reported. The proportion of genetic variation explained by the first two PCs was higher for GBS than for DArT markers, cumulatively explaining 14.4% and 8.9% of variation, respectively (**Figure 2**). The first five GBS PCs cumulatively explained 23.2% of variation while the equivalent DArT PCs explained 16.9%. For both GBS and DArT markers, PCoA did not clearly discriminate between lines from different countries of origin (**Figure 2**) although some basic grouping by origin was observed with the DEU and GBR lines clearly separated and those of French origin overlaying the other two. Structure analysis based on GBS revealed that the panel could be split into K = 4 groups as inferred from the analysis of the *ad hoc* ΔK statistic (Evanno et al., 2005; **Supplementary Figure S3**). Only 92 of the varieties could not be placed into a single distinctive group. Similar to the results of PCoA, the groups were not discriminated by country of origin.

### Association Mapping Using GBS

**Figure 3:**
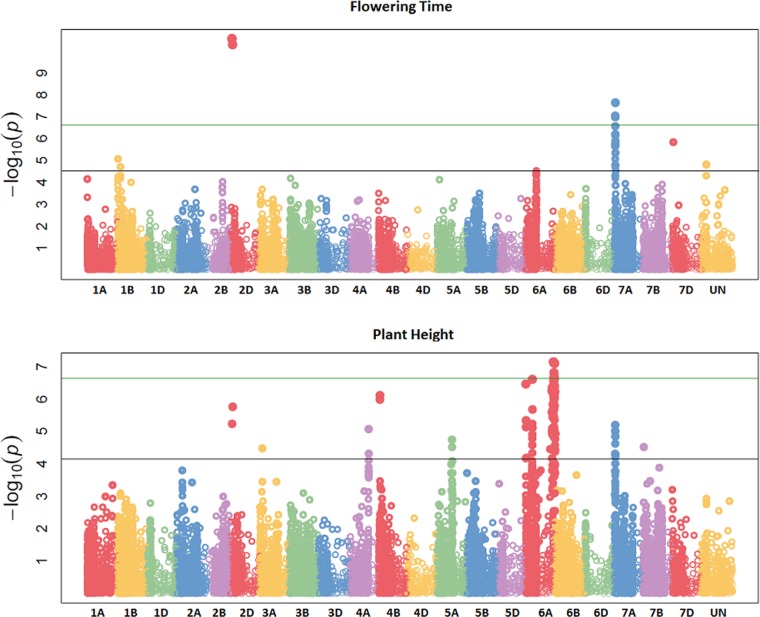
Manhattan plots summarizing association mapping results for flowering time (FT) and plant height (PH). The green line represents the experiment-wide Bonferroni adjusted threshold of p = 0.05 while the black line represents the false discovery rate (FDR) p-value of 0.05.

**Figure 4:**
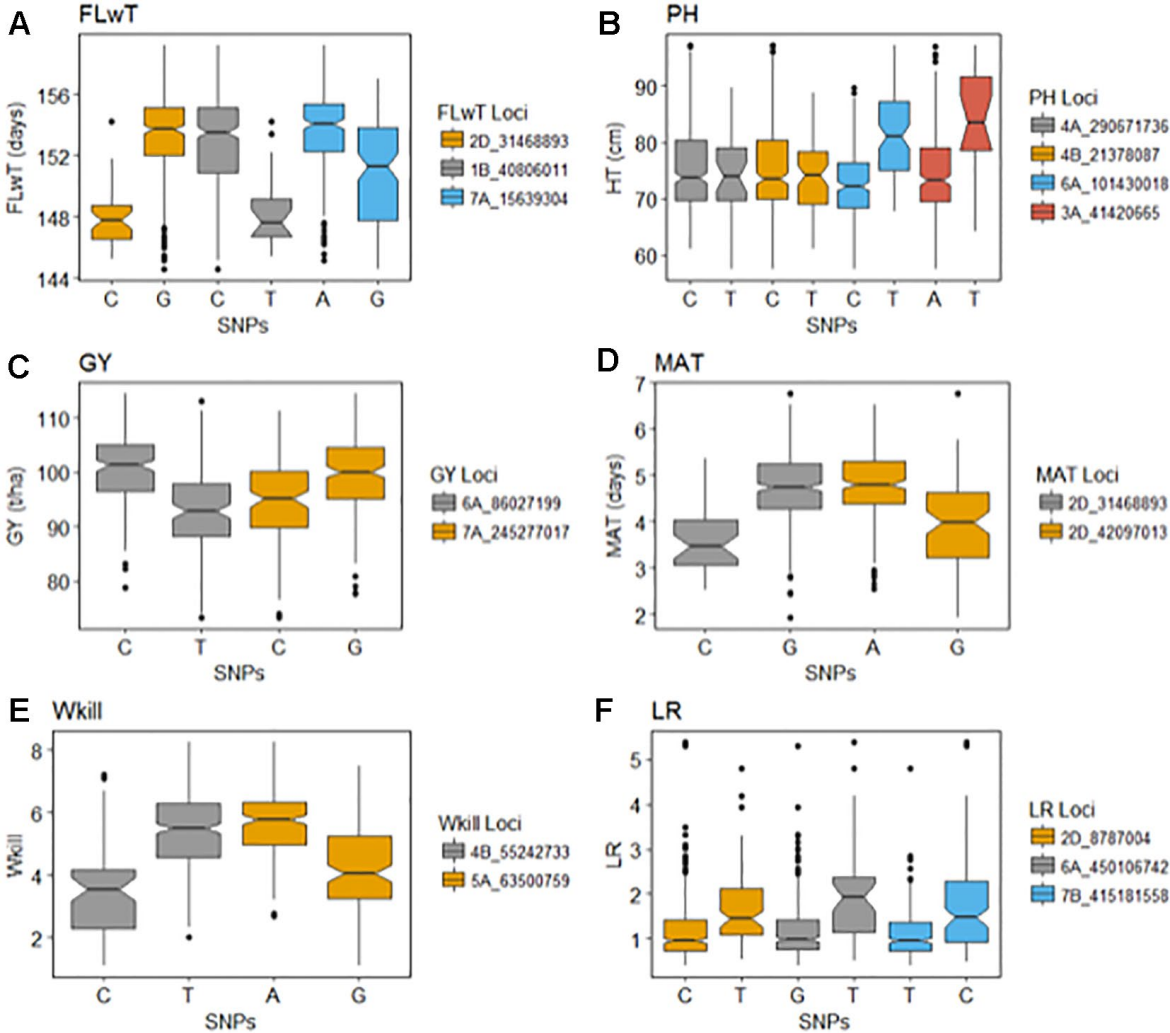
Effects of the selected significant alleles for flowering time (FT), plant height (PH), grain yield (GY), maturity (MAT), winter kill (WKill), and lodging resistance (LR).

GBS association mapping was conducted for GY, three yield-related (TGW, GSW, and ears/m²), seven morphological (FT, PH, Awns, LR, Wkill, TN, and MAT) and one quality (Gpt) trait using 42,795 SNPs and 350 individuals (**Supplementary Table S1**). The mixed model method detected a total of 63 loci (comprising 638 SNPs) with significant marker-trait associations for eight traits. Of the total number of significant SNPs, 77 were significant at the experiment-wide Bonferroni threshold (–log₁₀ p-value = 6.63) and the remaining 561 SNPs were declared significant at the less stringent FDR p-value ≤ 0.05. The total number of significant SNPs detected for each trait is shown in **Table 1**. No significant associations were detected for four traits; TN, TGW, GSW, and ears/m². Manhattan plots for FT and PH are presented in **Figure 3**. Manhattan plots for all other traits are in **Supplementary Figure S4**. For FT analysis across sites, significant marker-trait associations were detected on six chromosomes corresponding to eight loci and comprising of 47 SNPs (**Table 1**). Based on multiple regression analysis they together explained 45.1% of the variation in FT. Additional site-specific QTL were detected on chromosomes 2B in FRA (2010), 5A in DEU (2010) and 6D in FRA and DEU (2010). Two loci on chromosome 2D (**Figure 3**) (at physical positions 31468893 and 42097013) had the most significant association with FT at all sites controlling an average of 9.6 and 9.3% of FT variation, respectively (**Supplementary Table S3**). These two loci were presumed to be tightly linked with the *Ppd-D1* gene controlling photoperiod sensitivity. This was verified with the use of the *Ppd-D1* gene marker reported in Bentley et al. (2013) as a covariate which resulted in the loss of significant effect at the two loci. An environmentally stable QTL was also identified on 7A which encompassed up to 31 SNPs that controlled approximately 4% to 8% of phenotypic variation in FT (**Supplementary Table S3**). Two significant loci detected on chromosome 1B were more environment specific, only detected in the GBR and FRA in 2011 and 2010, respectively, and in the across site analysis (**Table 1**). The allelic effects for the three most significant SNPs on chromosomes 1B, 2D, and 7A are shown in the box plot summary in **Figure 4**. The 2D and 1B SNPs had alleles conferring the earliest flowering effect (147–148 days after planting) while the 7A SNP had a more intermediate effect (153–156 days after planting).

**Table 1:** Summary of quantitative trait loci (QTL) detected with significant marker-trait associations for across site and site-specific analysis for flowering time (FT) and grain yield (GY).

Trait	Site	Chromosomes	Number of loci	Number of SNPs	Range of variation controlled	Overall variation controlled (%)
**FT**	Across sites	1B, 2D, 6A, 7A, 7D, Un	8	47	3.4–9.6	45.10
	GBR 2010	2D, 7A, 7D	4	22	4.0–5.3	
	GBR 2011	1B, 2D, 7A	4	7	6.9–11.7	
	FRA 2010	1B, 2B, 2D, 6D, 7A, 7D, Un	8	31	3.8–8.8	
	FRA 2011	2D, 7A, Un	4	11	4.2–12.6	
	DEU 2010	2D, 5A, 6D, 7A, 7D, Un	7	39	3.7–7.6	
	DEU 2011	–	–	–	–	
GY*	Across sites	6A, 7B	2	13	3.1–3.9	32.98
	GBR 2010	–	–	–	–	
	GBR 2011	6A, 7A	2	13	3.3–4.1	
	FRA 2010	6A, 7B	2	10	3.4–4.0	
	FRA 2011	–	–	–	–	
	DEU 2010	–	–	–	–	
	DEU 2011	2A, 6A, 7B	3	9	4.1–5.0	
**PH**	Across sites	2D, 3A, 4A, 4B, 5A, 6A, 7A, 7B	12	123	2.5–5.0	52.55
**Awns**		1A, 1B, 1D, 2B, 2D, 3A, 4A, 4B, 5A, 5B, 6A, 6D, Un	13	147	3.0–28.9	76.92
**Wkill**		4B, 5A	2	16	3.6–4.6	33.35
						
**MAT**		2D	2	2	6.1–7.2	25.27
**Gpt**		1A, 3B, 4A, 5B, 6A, 6B, 7A, 7B	10	159	2.7–5.5	52.60

*QTL detected with the most significantly associated SNPs with PH and FT included in mixed model as covariate.

The number of loci identified, the total number of single nucleotide polymorphisms (SNPs) identified per loci, and the overall variation controlled based on mixed linear modeling are also shown.

GY QTL were detected on chromosomes 6A and 7B for the across site analysis when the most significant PH and FT SNPs (on 2D and 6A, respectively) were included as covariates in the mixed model. The same loci on 6A and 7B were also detected in the FRA (2010) and GBR (2011) experiments. Both SNPs produced equivalent effects on grain yield (**Figure 4**). Additional QTL were detected on chromosomes 2A and 7A from the DEU and GBR (2011) experiments, respectively (summarized in **Supplementary Table S3**). In total, 13 significant SNPs were found in association with GY, explaining 33% of variation (**Table 1**). Significant associations were detected for PH on eight chromosomes across all experiments comprising 12 loci and 123 SNPs. Together the SNPs explained approximately 53% of the total PH variation. The most significant QTL for PH was detected on chromosome 6A (**Figure 3**) (from physical position 373461190 to 452372111) with 76 SNPs (**Supplementary Table S3**) which controlled approximately 23% of the variation in PH. Two additional loci were also detected on chromosome 6A in association with PH (**Supplementary Table S3**). Covariate analysis with two of the most significant SNPs on 6A as fixed effects in the mixed model did not reveal any additional associations with PH. Significant QTL were also detected for PH on chromosomes 4A and 4B with FDR significance value ≤ 0.005 and 0.001, respectively. The previously detected FT QTL on 2D (31468893 and 42097013) were also significant for PH (FDR p-value ≤ 0.004 and 0.001, respectively).

Our analysis detected 147 SNPs in significant association with the presence/absence of awns across 13 chromosomes (**Supplementary Table S3**) which together controlled approximately 77% of variation (**Table 1**). The most significant QTL for presence/absence of awns was detected on chromosome 5A, comprising 71 SNPs which altogether controlled approximately 69% of the phenotype variation. The SNP with the highest significance at position 255590080 on 5A controlled approximately 29% of variation (**Supplementary Table S3**). Two QTL were detected in significant association with Wkill on chromosome 4B and 5A covering 16 SNPs which in total controlled 33 % of variation (**Table 1**). MAT was linked in association with FT on chromosome 2D (position 31468893 and 42097013) and together they explained approximately 25% of the variation. Significant associations were detected on eight chromosomes for Gpt comprising 10 loci of 159 SNPs which together explained 52% of variation controlled (**Table 1**). LR was significantly associated with 14 loci across 10 chromosomes, covering 128 SNPs which altogether explained over 50% of the variation present. The most significant QTL for LR, Gpt, PH, and GY colocalized within the same region of chromosome 6A (physical position 373461190 to 450106742) (**Supplementary Table S3**).

### Comparison of GBS and Dart Marker Mapping

**Figure 5:**
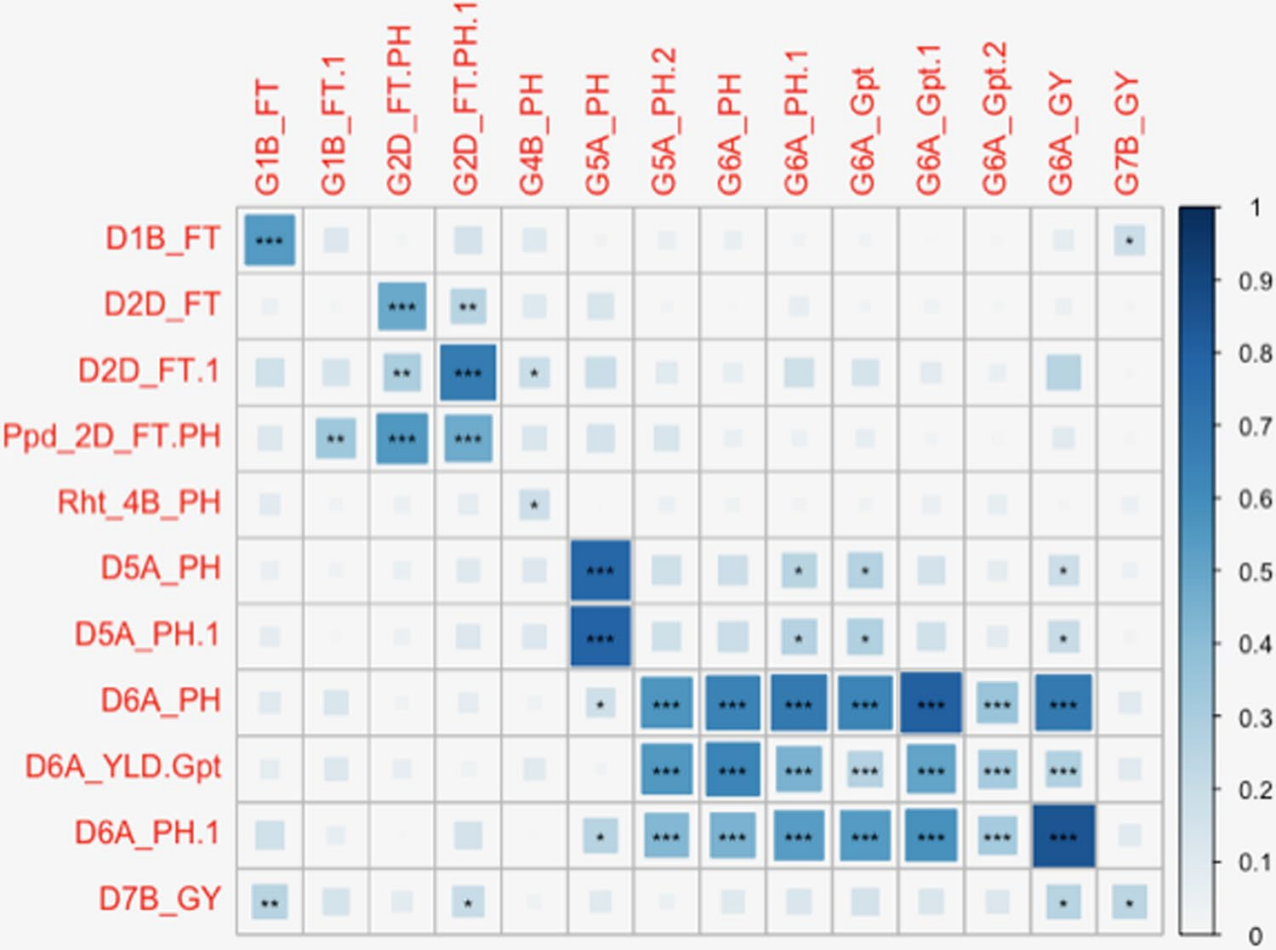
Diagrammatic representation of correlations between significant markers from Diversity Array Technology (DArT) and genotyping-by-sequencing (GBS) marker platforms. Significant DArT and GBS markers are shown on the vertical and horizontal axis respectively. The DArT and GBS markers used in the correlation shown here are significant for FT, PH, grain protein content (Gpt), and GY on chromosomes 1B, 2D, 4B, 5A, 6A, and 7B. The full names of markers used in correlation are shown in **Supplementary Table S6**. The size and shade of the squares corresponds to the magnitude of the correlation coefficient as shown in the scale. The p-values of correlations are as follows: p ≤ .05*, p ≤ .01**, p ≤ .001***.

The full panel of 376 lines had previously been genotyped with genome-wide dominant DArT markers and candidate adaptation gene markers with significant marker-trait associations detected for FT, GY, PH, Wkill, Gpt, and TGW (Bentley et al., 2014). These were compared to the GBS mapping results for QTL that had been detected on common and unique chromosomes (**Table 2**). **Figure 3** summarizes QTL detected using DArT for FT and PH. Pearson’s correlations of DArT and GBS markers significant for FT, GY, PH, and Gpt revealed highly significant correlations (P-value ≤ 0.001) between markers identified on common chromosomes (1B, 2D, 5A, and 6A) for the same traits (**Figure 5**; **Supplementary Table S6**). This is likely to be an indication that the significant loci were linked between the different marker platforms. GBS mapping detected more significant marker-trait associations (on more chromosomes) than DArT markers for FT, PH, and Gpt, but no significant association was detected for TGW and fewer QTL were identified for GY using GBS. Wkill QTL were identified on different chromosomes in the GBS compared to DArT mapping. In the present study no marker-trait associations were detected on chromosome 4D. GWAS analysis accounting for the *Ppd-D1* gene marker as a covariate resulted in loss of the two loci detected on chromosome 2D with GBS markers.

**Table 2:** Comparison of genotyping-by-sequencing (GBS) and Diversity Array Technology (DArT) mapping analysis based on the chromosomes on which significant associations were detected.

	Unique QTL		Common QTL
Traits	GBS	DArT	GBS & DArT
FLT	6A, 7A, 7D, Un	7B	1B, 2D
GY	5D	1B, 3A, 4A, 4D	6A,7B
PH	2B, 3A, 3B, 4A	4D	2D,4B, 5A, 6A
			
Wkill	5A,4B	2D, 6B	–
Gpt	1A, 4A, 5B, 6B,7A, 7B	–	6A
TGW	–	2B	–

### Genomic Prediction

**Figure 6:**
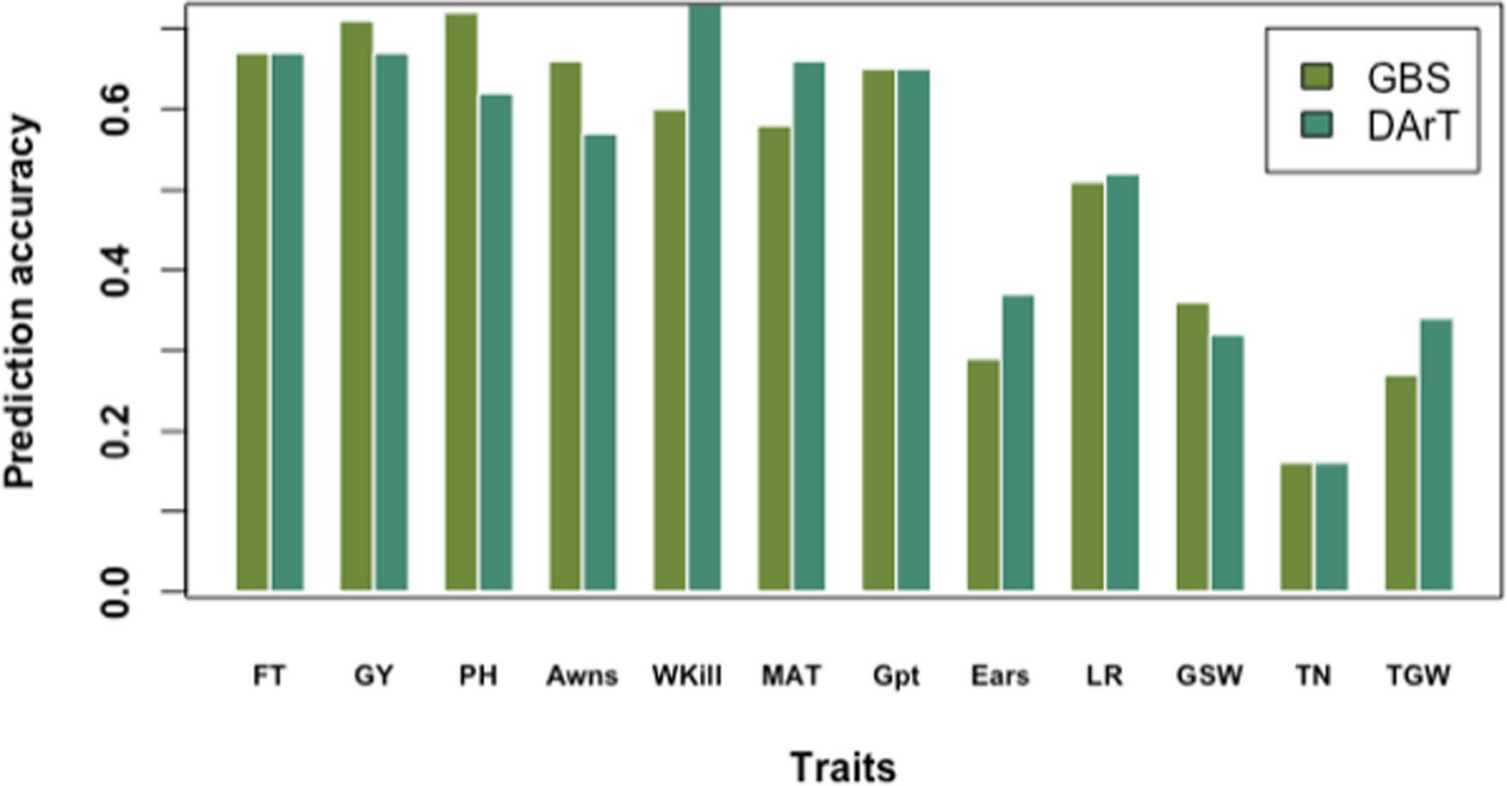
Prediction accuracy of genotyping-by-sequencing (GBS) versus Diversity Array Technology (DArT) markers based on tenfold cross-validation on the full data panel.

The highest prediction accuracies were observed for GY and PH, and the lowest for TN using cross-validation across the full panel (**Figure 6**). Prediction accuracies were highest for most of the traits when cross-validation was run across the full germplasm panel (rather than by country subsets). This trend was observed for most of the traits except Wkill which was predicted with highest accuracy in the DEU subset (**Supplementary Table S4**). The lowest accuracy values were recorded for the smallest population size in the GBR subset (**Supplementary Table S4**). In contrast, the highest accuracies were observed in FRA where the training population size was largest (**Supplementary Table S4**). Across country predictions, achieved by training the model on the subset of varieties from one country and predicting the values for the varieties from remaining two countries both singly and jointly also revealed the influence of training population size and degree of phenotypic variation. Accuracy was highest when FRA was used as the training population to predict GY in DEU, GBR and the combined DEU and GBR dataset and lowest when the GBR set was used as the training population to predict performance of the FRA and DEU sets (**Supplementary Table S5**). FT and Awns could only be predicted within the FRA varieties; while ears/m² was only predicted when the FRA varieties were used to train the prediction model.

Using GBS markers, prediction accuracies by tenfold cross-validation on the whole panel were higher than predictions in FRA, DEU, and GBR subsets for FT, PH, MAT, Gpt, LR, and GSW. A similar trend was observed using dart markers and cross-validation on the whole germplasm panel for predicting FT, PH, Wkill, MAT, and GSW (**Figure 6**). Overall predictions made using GBS by tenfold cross-validation for the full dataset resulted in higher genomic prediction accuracy for GY (0.71) compared to DArT markers (0.67). However, variation was observed for GY predictions by country and GBS gave higher predictions (Compared to DArT) in DEU, equivalent predictions in FRA and lower predictions in GBR. Predictions for FT on the whole panel were equivalent for both GBS and DArT markers. Predictions for Wkill, MAT, Ears, LR, and TGW revealed higher accuracy with DArT than with GBS markers both in cross-validation and training model experiments (**Figure 6**).

## Discussion

GBS is a genotyping tool combining simultaneous *de novo* sequencing and polymorphism discovery. It is useful for diverse variety panels, such as used in this study, to generate markers with broad potential relevance to breeding programs. In this study, a total of 42,795 SNPs were used to generate a high-density physical map and used to study the pattern of LD decay within the wheat genome. Our results showed that on average, LD decayed at the slowest rate on the D-genome and fastest on the A-genome while the B-genome had the largest proportion of polymporphic loci. A previous study of LD among several winter and spring wheat breeding populations revealed a similar pattern of decay among genomes for all the populations (Chao et al., 2010). This trend has been attributed to the latest polyploidization event between tetraploid (AABB) and diploid (DD) progenitors which gave rise to domesticated hexaploid bread wheat (Akhunov et al., 2010). Per chromosome, LD decayed fastest on 1B with the slowest rates recorded for chromosomes 1D, 2B, 2D, and 3D. This could be the result of indirect selection for blocks within these chromosomes containing genes conferring agronomic advantage within our collection of elite European varieties although there is not yet gene-level information to support this. The D-genome also had the lowest number of GBS SNPs with only 240 mapped to 4D and no QTL identified on this chromosome. Similarly, no QTL were identified on chromosomes 1D, 3D, and 5D thought to be generally indicative of the low levels of diversity in the D-genome (Akhunov et al., 2010).

Population structure analysis revealed that there was no clear structural partitioning within our association mapping panel. As the panel was assembled from elite lines originating from three different European countries, it was expected that the panel would be structured by country. Although the varieties in the panel did tend to approximately group by country of origin, there was no clear separation of clusters into country of origin. This is an indication of the extent to which European wheat breeding materials are related and exchanged among breeders. Similar trends were also observed in other studies on European winter wheat mapping panels (Langer et al., 2014; Albrecht et al., 2015). PCs derived from GBS markers explained a larger proportion of the variation that DArT markers, likely an effect of the larger number of markers available.

High density genotyping appreciably increased the precision of association mapping in the panel. This was established by the identification of similar loci to the previous study on the panel (Bentley et al., 2014) in addition to detection of loci not previously found. High density genetic linkage maps are one of the key factors for high precision QTL detection in association mapping studies (Elshire et al., 2011; Poland et al., 2012b). Both DArT and GBS use the methylation sensitive restriction enzyme *Pst1* for cutting the genome (with GBS also using methylation sensitive *Msp1* as the second cutting enzyme while DArT uses nonmethylation sensitive *MseI*). In combination with the diverse panel of germplasm used in this study, we expected that as a result SNPs in strong LD with known genes and causal loci should be detected to a high precision, and to a higher degree with GBS compared to DArT. Although some DArT markers are anchored to RefSeq v1.0 and are available *via* the Wheat@URGI portal (Alaux et al., 2018) it is not currently possible to anchor the DArT markers used in Bentley et al., 2014 to the physical map to facilitate a complete comparison of QTL detection. While previous studies have shown that it is possible to find microsatellite repeats within DArT microarray clone sequences and then design PCR-based markers and assign to map locations, this is a low-throughput process (Fiust et al, 2015). However, we are able to report on the scale and correlation of detected marker trait associations and predictive ability between the anchored GBS data and previous DArT data.

Marker trait associations for FT were identified at seven loci across five chromosomes. Three of the associations detected for FT mapped to chromosomes with known genes regulating FT. The two loci detected on chromosome 2D were established to be linked to *Ppd-D1* (Beales et al., 2007) when the gene marker was accounted for as a covariate in the mixed model. Chromosomes 1B and 7A have both been associated with *Earliness per se* in separate studies by Griffiths et al. (2009) and Hanocq et al. (2004). Chromosome 7A carries the vernalization gene, *Vrn-3A*, which accelerates flowering in wheat. The two QTL on 2D and the locus on 7A (spanning 4 to 31 SNPs) were also stable across all trial environments. They reveal good potential for FT genetic marker screening in breeding materials and variability in allelic effects for these SNPs can be potentially useful in marker assisted breeding where these loci are not already fixed.

PH QTL were detected across 12 loci on eight chromosomes. The most significant QTL on 6A (373461190:452372111) was also identified to be highly significant in a QTL mapping study in a RIL population by Marza et al. (2006). In the previous GWAS study with DArT markers (Bentley et al., 2014), the most significant PH QTL was the *Rht-D1* gene marker on chromosome 4D. No QTL were mapped with GBS to chromosome 4D in this study. This is in contrast to our expectation that GBS should detect known genes to high precision and is likely to be due to a lack of SNPs in sufficient LD with this gene; only 240 GBS SNPs were identified on 4D (**Supplementary Table S2**). However, two loci were identified to be associated with PH on chromosomes 4A (290527503:291878645) and 4B (21378087:21379808) which may be linked to homologues of *Rht-D1* on 4D (18780696:18781314) (Worland et al., 1998; Wilhelm et al., 2013) although there is not a correspondence in physical position. Although a homoeologous locus (*Rht-A1*) exists on 4A, and has been shown to express the DELLA protein, linked markers, or phenotypic effects on plant height have yet to be determined (Pearce et al., 2011).

**Figure 7:**
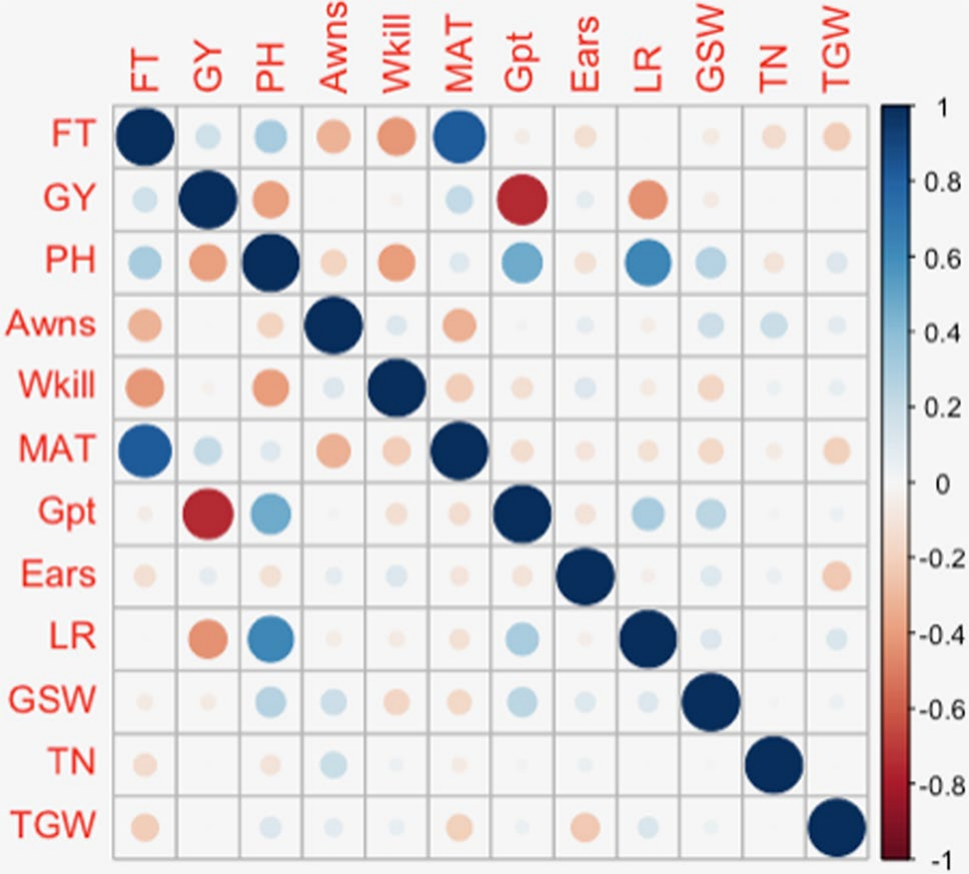
Diagrammatic representation of Pearson’s pairwise correlation of phenotype values.

The presence or absence of awns is a simple trait controlled by a known locus with a large effect on 5A (Kato et al., 1998; Mackay et al., 2014). In this study GBS detected this major effect locus in the same location as the previously validated marker tagging the 5AL genetic locus (previously reported based on validation in this panel). Additional minor QTL linked with the presence/absence of awns were detected on twelve other chromosomes. Although it is a binary trait (presence/absence), these additional QTL could be useful to understanding the genetic network controlling the presence of awns. Further understanding of the genetic architecture of this trait is relevant to breeding as awns have been shown to contribute to photosynthesis and increase in grain size and yield in drought stressed environments (Rebetzke et al., 2016). Both environmentally stable and site-specific QTL were identified for GY. The QTL on 6A was detected in all three European trials. Other QTL and association mapping studies have also reported loci on 6A associated with yield under varying environmental conditions (Cui et al., 2014; Edae et al., 2014; Sukumaran et al., 2014). Chromosomes 2D and 6A featured associations with several key traits. Six traits: FT, GY, PH, Awns, Gpt, and LR were identified in association with loci on 6A while FT, PH, MAT, and LR were associated with loci on 2D. Similar to this trend, Marza et al. (2006) also observed the colocalization of QTL for several traits on chromosome 6A. Most of the traits with colocalized QTL in the present study were also observed to have high positive or negative correlations with each other (**Figure 7**). A similar pattern was observed in an association study of QTL controlling agronomic traits in an elite rice breeding panel (Begum et al., 2015). On chromosome 2D, the same SNPs were found in significant association with FT, PH, and MAT while a nearby SNP was found in association with LR. SNPs on 6A, significantly associated with GY, PH, Gpt, and LR were located within the same region of the chromosome and were in LD. This observation supports the likelihood of pleiotropy on 2D and an underlying gene linkage on 6A. Photoperiod insensitivity and reduced height genes have a positive impact on GY and LR (Hedden, 2003; Wilhelm et al., 2013). Reduced height and photoperiod genes have been shown to enhance LR while simultaneously conferring adaptive advantages to favor GY in different agro-climatic conditions (Worland et al., 1998; Donmez et al., 2001). The overlap between GY and Gpt loci on 6A could potentially be exploited to simultaneously improve yield and quality traits of wheat. Phenotype correlations between GY and Gt were however observed to be highly negative (r² = –0.75) (**Figure 7**). A similar negative association was found in a QTL mapping study of GY and grain quality by Tsilo et al. (2010) and has also been reviewed in detail by Balyan et al. (2013). Breeding efforts to increase Gpt resulted in lower genetic gains in yield compared to high yielding cultivar checks. Several QTL have been detected in this study for Gpt which are possibly independent of GY that could be further studied for exploitation in breeding.

Comparison of predictive abilities between the two marker platforms for all traits revealed only slight differences in accuracies with predictive ability mostly depending on the genetic architecture of the trait. Using GBS, GY was predicted with higher accuracy than FT despite fewer loci detected in significant marker trait association with GY. This could indicate the effectiveness of GBS in GS for capturing many small effect loci underlying GY which did not reach the significance threshold for association mapping. On the other hand, FT was predicted with greater accuracy using DArT markers in most of the scenarios tested with the highest accuracy estimated within the FRA variety set. Prediction of FT in the DEU and GBR germplasm was ineffective. As discussed by Bentley et al. (2014), this was due to the dominating effect of *Ppd-D1a* photoperiod insensitive mutation within the FRA germplasm which conferred earlier flowering effects for the FRA varieties compared to those from DEU and GBR which are almost exclusively photoperiod sensitive *Ppd-D1b* types. Due the absence of variation for awns within the DEU and GBR germplasm, awn presence or absence could not be predicted within the country subsets by tenfold cross-validation.

In a similar study by Poland et al. (2012a), GBS consistently produced higher prediction accuracies than DArT markers for 1,000 kernel weight and heading date, even with a comparable number of GBS and DArT markers. Jiang et al. (2015) conducted GS for prediction of resistance to Fusarium Head Blight using three different marker platforms (single sequence repeats, a 9K SNP array, and a 90K SNP array) and observed similar prediction accuracies with the three platforms for three prediction models. They concluded that relatedness was a key driver of prediction accuracy and we propose that the ability of the higher density of GBS markers to account for kinship is the main driver for increased prediction accuracies in this study. Validation of GS in diverse germplasm is important for the integration of this method in routine breeding programs. As shown in this study, GS across country germplasm is feasible for most of the traits measured, however, the composition of the training populations needs to be optimized for adequate genetic variation.

## Conclusion

The use of GBS has potential for practical application in wheat breeding and is a cost-effective platform for generating thousands of polymorphic SNPs with genome-wide coverage. Using the IWGSC Ref Seq v1.0 (International Wheat Genome Sequencing Consortium, 2018) for alignment of sequence reads and variant SNP calling enabled the generation of over 40,000 high-quality SNP data points. When applied to association mapping and genomic prediction in European winter wheat, GBS data anchored to IWGSC RefSeq v1.0 generally improved accuracy. In particular, this study demonstrates the utility of GBS for effectively predicting traits with many loci of small effects proving its suitability for GS. For mapping, the high marker density provided by GBS enhanced the precision of QTL mapping by increasing the probability of finding and tagging causal polymorphisms, although this was still limited on the D-genome. Prediction accuracies were higher when calculated across the panel; however, accuracy was highly dependent on the trait genetic architecture. This feature was common across both GBS and DArT marker platforms.

## Author Contributions

AB designed and oversaw the experiments. OL and IM conducted the analysis. JP generated GBS data and oversaw sequence anchoring and variant calling. SP coordinated the collection of new field phenotyping data. JH contributed to analysis and interpretation of data. OL wrote the paper. All authors contributed reviewing the manuscript.

## Funding

We acknowledge the support for Olufunmilayo Ladejobi’s PhD through the Biotechnology and Biological Sciences Research Council (BBSRC) and Department for International Development (DfID) Sustainable Crop Production Research for International Development (SCPRID) project “Wild Rice MAGIC” led by JH (BB/J011754/1). AB is supported by the BBSRC Cross-Institute Strategic Programme “Designing Future Wheat” BB/P016855/1. AB and JH are supported by the GCRF GROW project TIGR2ESS (BB/P027970/1).

## Conflict of Interest

The panel and phenotypes described were generated as part of the European Commission grant under the 7 Framework Programme for Research and Technological Development (FP7-212019). The funders had no role in the design or analysis of the experiments presented. The authors declare that the research was conducted in the absence of any commercial or financial relationships that could be construed as a potential conflict of interest. Author Ian J Mackay was employed by company IMPlant Consultancy Ltd. Author Sebastien Praud was employed by company Biogemma. All other authors declare no competing interests.
