# Supplementary material for: Reference Genome Anchoring of High-Density Markers for Association Mapping and Genomic Prediction in European Winter Wheat
Source: Front Plant Sci. 2019 Nov 8;10:1278. doi: 10.3389/fpls.2019.01278 (PMC6857554; doi:10.3389/fpls.2019.01278)

Supplementary Tables

**Table S1.** Summary of phenotypic traits

| **Traits** | **Method of data collection** |
| --- | --- |
| Flowering time (FT) | Days to 50 % ear emerged from the flag leaf (GS55; Zadoks et al. 1974). |
| Grain yield (GY) | t/ha at 15 % moisture |
| Plant height (PH) | Length in centimetres from stem base to top of spike at maturity. |
| Awns | 1 for presence of awn and 0 for absence |
| Winter kill (Wkill) | Visual scale of 1 – 9 from 1=low damage to 9=heavily damaged |
| Maturity (MAT) | number of days from ear emergence to maturity |
| Grain protein content (Gpt) | measured using an InfraTec system |
| Ears | Number of wheat ears in a square metre quadrant |
| Lodging resistance (LR) | The angle of inclination of the stem base from the vertical and the lodged area of the whole plot (calculated as percent of plot area lodged × angle of lodging from vertical position/90) |
| Grain specific weight (GSW) | grain hectolitre weight |
| Tiller number (TN) | Number of Tillers at maturity |
| Thousand grain weight (TGW) | Weight of 1000 grains measured using an InfraTec system |

| **Table S2**: Decay distance of LD at r² = 0.2 and the number and corresponding proportion of SNPs on each chromosome. The last row ‘UN’ is the number of SNPs in the unmapped group | | | |
| --- | --- | --- | --- |
| Chromosome | Number of SNPs | % of SNP markers | LD decay dist (Mbp) |
| 1A | 2062 | 4.82 | 2.0 |
| 2A | 2556 | 5.97 | 3.0 |
| 3A | 1857 | 4.34 | 3.0 |
| 4A | 1097 | 2.56 | 5.0 |
| 5A | 2175 | 5.08 | 5.0 |
| 6A | 2033 | 4.75 | 5.0 |
| 7A | 3439 | 8.04 | 5.0 |
| 1B | 4878 | 11.40 | 1.0 |
| 2B | 1918 | 4.48 | 10.0 |
| 3B | 3794 | 8.87 | 2.5 |
| 4B | 1686 | 3.94 | 5.0 |
| 5B | 2905 | 6.79 | 5.0 |
| 6B | 3663 | 8.56 | 3.0 |
| 7B | 3455 | 8.07 | 5.0 |
| 1D | 708 | 1.65 | 10.0 |
| 2D | 1176 | 2.75 | 10.0 |
| 3D | 611 | 1.43 | 10.0 |
| 4D | 240 | 0.56 | 2.5 |
| 5D | 606 | 1.42 | 5.0 |
| 6D | 448 | 1.05 | 5.0 |
| 7D | 554 | 1.29 | 2.5 |
| UN* | 934 | 2.18 | na |

| **Table S3.**  List of all QTL identified by site and location. The physical positions of QTL from start to end position of the loci are shown with the position of the most significant SNP, its minor allele frequency, P-value, and percentage variation. | | | | | | | | | | |
| --- | --- | --- | --- | --- | --- | --- | --- | --- | --- | --- |
| **Traits** | **Site** | **Chromosome** | **SNP start site** | **SNP end site** | **Total SNPs** | **Percentage variation explained** | **Most significant SNP** | **P-value** | **FDR P-value** | **MAF** |
| **FT across sites** | | 2D | 31468893 |  | 1 | 9.6 | 31468893 | 2.77E-11 | 1.14E-06 | 0.12 |
|  |  | 2D | 42097013 |  | 1 | 9.3 | 42097013 | 5.33E-11 | 1.14E-06 | 0.26 |
|  |  | 1B | 4341220 |  | 1 | 4.1 | 4341220 | 8.72E-06 | 0.014924 | 0.12 |
|  |  | 1B | 40429328 | 40806011 | 4 | 3.4 - 3.7 | 40806011 | 1.99E-05 | 0.025019 | 0.06 |
|  |  | 6A | 161648992 | 162945233 | 5 | 3.4 - 3.6 | 162222186 | 2.99E-05 | 0.035549 | 0.06 |
|  |  | 7A | 14418771 | 22872792 | 31 | 3.4 -6.6 | 15639304 | 2.28E-08 | 0.000244 | 0.35 |
|  |  | 7D | 16704573 |  | 1 | 4.9 | 16704573 | 1.52E-06 | 0.004414 | 0.28 |
|  |  | Un | 75187410 | 75249242 | 3 | 4.3 - 4.8 | 75248474 | 1.49E-05 | 0.021956 | 0.08 |
| **FT GBR 2010** | | 2D | 31468893 |  | 1 | 4.7 | 42097013 | 1.37E-06 | 0.013423 | 0.26 |
|  |  | 2D | 42097013 |  | 1 | 5.1 | 31468893 | 3.65E-06 | 0.013423 | 0.12 |
|  |  | 7A | 14418771 | 15997645 | 19 | 4.0 - 5.3 | 15639304 | 1.61E-06 | 0.013423 | 0.35 |
|  |  | 7D | 16704573 |  | 1 | 4.6 | 16704573 | 4.60E-06 | 0.013423 | 0.28 |
| **FT GBR 2011** | | 1B | 40806011 |  | 1 | 3.8 | 40806011 | 6.81E-06 | 0.041631 | 0.06 |
|  |  | 2D | 42097013 |  | 1 | 9.1 | 42097013 | 9.91E-12 | 4.24E-07 | 0.26 |
|  |  | 2D | 31468893 |  | 1 | 6.9 | 31468893 | 2.28E-09 | 4.87E-05 | 0.12 |
|  |  | 7A | 14418771 | 15639306 | 4 | 3.9-4.3 | 14455568 | 1.64E-06 | 0.023372 | 0.26 |
|  | |  |  |  |  |  |  |  |  |  |
| **FT FRA 2010** | | 1B | 4341220 |  | 1 | 4.7 | 4341220 | 5.45E-06 | 0.014961 | 0.12 |
|  |  | 2B | 235214268 | 235214298 | 4 | 4.0 | 235214268 | 2.77E-05 | 0.039551 | 0.08 |
|  |  | 2D | 31468893 |  | 1 | 8.8 | 31468893 | 8.06E-10 | 3.45E-05 | 0.12 |
|  |  | 2D | 42097013 |  | 1 | 8.1 | 42097013 | 3.36E-09 | 7.18E-05 | 0.26 |
|  |  | 6A | 162064175 |  | 1 | 3.8 | 162064175 | 3.60E-05 | 0.049693 | 0.05 |
|  |  | 7A | 14418771 | 15997645 | 20 | 4.1 - 6.5 | 15639304 | 1.08E-07 | 0.001157 | 0.35 |
|  |  | 7D | 16704573 |  | 1 | 5.1 | 16704573 | 2.39E-06 | 0.010243 | 0.28 |
|  |  | Un | 75187410 | 75248474 | 2 | 4.2 | 75248474 | 1.51E-05 | 0.028068 | 0.08 |
| **FT FRA 2011** | | 2D | 31468893 |  | 1 | 12.6 | 31468893 | 2.04E-13 | 8.71E-09 | 0.12 |
|  |  | 2D | 42097013 |  | 1 | 10.4 | 42097013 | 1.67E-11 | 3.57E-07 | 0.26 |
|  |  | UN | 75187410 |  | 1 | 4.2 | 75187410 | 1.25E-05 | 0.049407 | 0.08 |
|  |  | 7A | 14418771 | 178473942 | 8 | 4.2 - 5.6 | 15639304 | 5.30E-07 | 0.005675 | 0.35 |
| **FT GER 2010** | | 2D | 31468893 |  | 1 | 7.4 | 31468893 | 1.07E-08 | 0.000109 | 0.12 |
|  |  | 2D | 42097013 |  | 1 | 7.1 | 42097013 | 1.78E-08 | 0.000109 | 0.26 |
|  |  | 5A | 255590080 |  | 1 | 3.9 | 255590080 | 2.59E-05 | 0.031663 | 0.07 |
|  |  | 6D | 11414393 | 11794749 | 2 | 4.0 - 4.5 | 11414393 | 5.71E-06 | 0.008102 | 0.23 |
|  |  | 7A | 14418771 | 20287559 | 31 | 3.7 - 7.6 | 15639304 | 5.58E-09 | 0.000107 | 0.36 |
|  |  | 7D | 16704573 |  | 1 | 5.7 | 16704573 | 3.77E-07 | 0.000768 | 0.28 |
|  |  | Un | 75187410 | 75248474 | 2 | 3.8 | 75187410 | 2.90E-05 | 0.033508 | 0.08 |
| **GY across sites** | | 6A | 85580220 | 91681786 | 11 | 3.1 - 3.9 | 88260960 | 1.37E-06 | 0.02215 | 0.39 |
|  |  | 7B | 63134685 | 63170244 | 4 | 3.3 - 3.7 | 63134685 | 2.80E-06 | 0.02215 | 0.06 |
| **GY GBR 2011** | | 6A | 85580220 | 91330138 | 12 | 3.3 - 4.1 | 86027199 | 1.23E-06 | 0.022681 | 0.38 |
|  |  | 7A | 245277017 |  | 1 | 3.4 | 245277017 | 8.79E-06 | 0.044466 | 0.39 |
| **GY FRA 2010** | | 6A | 85580220 | 91681786 | 7 | 3.4 - 4.0 | 91681786 | 3.49E-06 | 0.043669 | 0.44 |
|  |  | 7B | 63170228 | 63170244 | 3 | 3.8 | 63170228 | 6.70E-06 | 0.043669 | 0.06 |
| **GY DEU 2011** | | 2A | 286621567 |  | 1 | 4.0 | 286621567 | 9.14E-07 | 0.018297 | 0.06 |
|  |  | 6A | 91330138 |  | 1 | 4.0 | 91330138 | 9.68E-06 | 0.047464 | 0.05 |
|  |  | 7B | 51065955 | 63170244 | 7 | 4.1 - 5.0 | 63134685 | 9.98E-06 | 0.047464 | 0.44 |
|  | |  |  |  |  |  |  |  |  |  |
| **PH** | | 2D | 31468893 |  | 1 | 3.5 | 31468893 | 5.74E-06 | 0.004074 | 0.12 |
|  |  | 2D | 42097013 |  | 1 | 3.9 | 42097013 | 3.29E-05 | 0.001821 | 0.05 |
|  |  | 3A | 41420665 |  | 1 | 2.9 | 41420665 | 3.29E-05 | 0.01514 | 0.05 |
|  |  | 4A | 290527503 | 291878645 | 4 | 2.7 - 3.4 | 290671736 | 8.40E-06 | 0.005137 | 0.09 |
|  |  | 4B | 21378087 | 21379808 | 2 | 4.2 | 21378087 | 7.58E-07 | 0.001207 | 0.18 |
|  |  | 5A | 212741140 | 232561580 | 4 | 2.7 - 3.1 | 226572725 | 1.83E-05 | 0.009087 | 0.34 |
|  |  | 6A | 893200 | 6018237 | 6 | 2.7 - 4.5 | 2234764 | 8.40E-06 | 0.005137 | 0.09 |
|  |  | 6A | 8993644 | 101851274 | 15 | 2.5 - 4.5 | 101430018 | 2.54E-07 | 0.001089 | 0.28 |
|  |  | 6A | 373461190 | 452372111 | 76 | 2.5 - 5.0 | 422756419 | 3.44E-07 | 0.001089 | 0.31 |
|  |  | 7A | 14418771 | 15750691 | 11 | 2.6 - 3.5 | 15031731 | 6.48E-06 | 0.004473 | 0.40 |
|  |  | 7B | 11703887 |  | 1 | 3.0 | 11703887 | 3.02E-05 | 0.014183 | 0.41 |
|  |  | 7B | 248997934 |  | 1 | 2.5 | 248997934 | 0.000132 | 0.046269 | 0.14 |
|  | |  |  |  |  |  |  |  |  |  |
|  |  |  |  |  |  |  |  |  |  |  |
| **Awns** |  | 1A | 61129013 | 61225489 | 4 | 3.4 -4.3 | 61225489 | 4.90E-06 | 0.002591 | 0.11 |
|  |  | 1B | 189392425 | 198075863 | 13 | 3.1 -9.6 | 189392425 | 1.81E-11 | 1.55E-07 | 0.05 |
|  |  | 1D | 343103677 |  | 1 | 3.2 | 343103677 | 7.76E-05 | 0.025338 | 0.11 |
|  |  | 2B | 337609921 | 347561440 | 3 | 3.5 - 3.7 | 341758577 | 2.29E-05 | 0.009425 | 0.06 |
|  |  | 2D | 7738369 |  | 1 | 2.9 | 7738369 | 0.00016 | 0.046633 | 0.10 |
|  |  |  |  |  |  |  |  |  |  |  |
|  | | 3A | 161444257 |  | 1 | 3.2 | 161444257 | 7.30E-05 | 0.025006 | 0.06 |
|  |  | 4A | 266558255 | 277317797 | 4 | 3.2 -3.5 | 266558255 | 8.00E-06 | 0.364923 | 0.10 |
|  |  | 4B | 140267912 |  | 1 | 3.3 | 140267912 | 5.94E-05 | 0.021914 | 0.06 |
|  |  | 5A | 227301584 | 256524929 | 71 | 3.0 - 28.9 | 255590080 | 4.56E-28 | 1.95E-23 | 0.07 |
|  |  | 5B | 129053488 | 137384512 | 14 | 3.0 -5.3 | 137384512 | 4.05E-07 | 0.000576 | 0.10 |
|  |  | 6A | 3758004 | 21719436 | 26 | 3.0 - 10.8 | 16462141 | 1.37E-12 | 1.46E-08 | 0.05 |
|  |  | 6D | 5155743 | 17349048 | 7 | 4.1 - 6.3 | 5155743 | 5.45E-07 | 0.000576 | 0.07 |
|  |  | Un | 71499496 |  | 1 | 3.7 | 71499496 | 1.86E-05 | 0.007852 | 0.05 |
| **Wkill** | | 4B | 55242733 |  | 1 | 3.7 | 63500759 | 1.15E-06 | 0.042022 | 0.37 |
|  |  | 5A | 59536488 | 67301668 | 15 | 3.6 - 4.6 | 55242733 | 1.03E-05 | 0.042797 | 0.18 |
|  | |  |  |  |  |  |  |  |  |  |
|  |  |  |  |  |  |  |  |  |  |  |
|  |  |  |  |  |  |  |  |  |  |  |
|  |  |  |  |  |  |  |  |  |  |  |
|  |  |  |  |  |  |  |  |  |  |  |
|  |  |  |  |  |  |  |  |  |  |  |
|  |  |  |  |  |  |  |  |  |  |  |
|  |  |  |  |  |  |  |  |  |  |  |
|  |  |  |  |  |  |  |  |  |  |  |
|  |  |  |  |  |  |  |  |  |  |  |
|  |  |  |  |  |  |  |  |  |  |  |
|  |  |  |  |  |  |  |  |  |  |  |
|  |  |  |  |  |  |  |  |  |  |  |
|  | |  |  |  |  |  |  |  |  |  |
|  |  |  |  |  |  |  |  |  |  |  |
| **MAT** |  | 2D | 31468893 |  | 1 | 6.1 |  | 1.51E-08 | 0.000647 | 0.12 |
|  |  | 2D | 42097013 |  | 1 | 7.2 |  | 1.63E-07 | 0.003486 | 0.26 |
| **Gpt** | | 1A | 42151448 |  | 1 | 2.9 | 42151448 | 0.000101 | 0.031923 | 0.40 |
|  |  | 3B | 5601689 |  | 1 | 2.7 | 5601689 | 0.000184 | 0.0497 | 0.29 |
|  |  | 4A | 186503286 | 277186559 | 5 | 2.7 - 3.1 | 186503286 | 4.83E-05 | 0.021768 | 0.14 |
|  |  | 5B | 107367824 | 171131833 | 2 | 2.7 - 3.8 | 107367824 | 5.62E-05 | 0.023814 | 0.38 |
|  |  | 6A | 85263886 | 115457176 | 130 | 2.7 - 5.5 | 112688966 | 9.91E-08 | 0.004054 | 0.23 |
|  |  | 6A | 151492080 |  | 1 | 2.8 | 151492080 | 9.38E-06 | 0.011145 | 0.11 |
|  |  | 6A | 416675591 | 448706137 | 8 | 2.7 - 3.8 | 448010707 | 0.000111 | 0.03349 | 0.36 |
|  |  | 6B | 106381266 |  | 1 | 3.9 | 106381266 | 6.25E-06 | 0.010774 | 0.19 |
|  |  | 7A | 62185902 |  | 1 | 2.8 | 62185902 | 0.000119 | 0.034994 | 0.39 |
|  |  | 7B | 50826708 | 63170244 | 9 | 2.7 - 3.7 | 63134685 | 1.14E-05 | 0.011576 | 0.06 |
|  |  |  |  |  |  |  |  |  |  |  |
|  |  |  |  |  |  |  |  |  |  |  |
| **LR** | | 1B | 1467644 | 204532429 | 3 | 3.5- 4.8 | 1467644 | 7.80E-06 | 0.013346 | 0.09 |
|  |  | 2A | 3634548 |  | 1 | 3.9 | 3634548 | 5.15E-05 | 0.044092 | 0.10 |
|  |  | 2D | 8787004 |  | 1 | 5.0 | 8787004 | 5.18E-06 | 0.013346 | 0.21 |
|  |  | 2D | 11051777 | 146583225 | 94 | 3.4 - 5.0 | 110701290 | 1.03E-05 | 0.013346 | 0.05 |
|  |  | 3A | 9460408 |  | 1 | 3.5 | 9460408 | 0.000122 | 0.045094 | 0.10 |
|  |  | 4A | 222595975 | 298655753 | 3 | 3.5 - 3.7 | 298655753 | 3.69E-05 | 0.033413 | 0.14 |
|  |  | 4B | 69072046 |  | 1 | 4.1 | 69072046 | 3.39E-05 | 0.032975 | 0.06 |
|  |  | 6A | 2234764 | 13200436 | 8 | 3.4 - 4.7 | 13200405 | 8.41E-06 | 0.013346 | 0.06 |
|  |  | 6A | 101430018 |  | 1 | 4.4 | 101430018 | 1.70E-05 | 0.019418 | 0.28 |
|  |  | 6A | 373461190 | 450106742 | 4 | 3.5 - 6.1 | 450106742 | 4.74E-07 | 0.013346 | 0.11 |
|  |  | 6D | 2449348 |  | 1 | 3.7 | 2449348 | 8.18E-05 | 0.045094 | 0.15 |
|  |  | 7A | 278811242 |  | 1 | 3.4 | 2.79E+08 | 0.000139 | 0.04717 | 0.08 |
|  | | 7B | 45162103 | 65376987 | 4 | 3.5 - 4.3 | 45162103 | 2.30E-05 | 0.022875 | 0.30 |
|  |  | 7B | 224812991 | 248997934 | 5 | 3.5 - 3.7 | 224812991 | 7.52E-05 | 0.045094 | 0.12 |
|  |  |  |  |  |  |  |  |  |  |  |

| **Supplementary Table S4.** Predictive correlations based on 10-fold cross validation for the full panel and the panel split by country for GBS versus DArT marker data (GBR =70, DEU = 82 and FRA = 192) | | | | | | | | | | | |
| --- | --- | --- | --- | --- | --- | --- | --- | --- | --- | --- | --- |
| **Traits** | **Full Dataset** | |  | **FRA** | |  | **DEU** | |  | **GBR** | |
|  | **GBS** | **DArT** |  | **GBS** | **DArT** |  | **GBS** | **DArT** |  | **GBS** | **DArT** |
| FT | 0.67 | 0.67 |  | 0.40 | 0.65 |  | NA | NA |  | NA | NA |
| GY | 0.71 | 0.67 |  | 0.71 | 0.70 |  | 0.73 | 0.63 |  | 0.47 | 0.68 |
| GY | 0.71 | 0.67 |  | 0.71 | 0.70 |  | 0.73 | 0.63 |  | 0.47 | 0.68 |
| PH | 0.72 | 0.62 |  | 0.59 | 0.64 |  | 0.56 | 0.46 |  | 0.68 | 0.60 |
|  |  |  |  |  |  |  |  |  |  |  |  |
| Awns | 0.66 | 0.57 |  | 0.79 | 0.58 |  | NA | NA |  | NA | NA |
| WKill | 0.60 | 0.73 |  | 0.39 | 0.27 |  | 0.78 | 0.78 |  | 0.12 | 0.02 |
| MAT | 0.58 | 0.66 |  | 0.37 | 0.51 |  | 0.19 | 0.07 |  | 0.33 | 0.55 |
| Gpt | 0.65 | 0.65 |  | 0.51 | 0.65 |  | 0.72 | 0.67 |  | 0.34 | 0.53 |
|  |  |  |  |  |  |  |  |  |  |  |  |
|  |  |  |  |  |  |  |  |  |  |  |  |
| Ears | 0.29 | 0.37 |  | 0.28 | 0.26 |  | 0.44 | 0.34 |  | 0.47 | 0.23 |
| LR | 0.51 | 0.52 |  | 0.51 | 0.45 |  | 0.39 | 0.21 |  | 0.55 | 0.35 |
| GSW | 0.36 | 0.32 |  | 0.30 | 0.30 |  | 0.46 | 0.53 |  | 0.16 | 0.12 |
|  |  |  |  |  |  |  |  |  |  |  |  |
| TN | 0.16 | 0.16 |  | 0.10 | 0.10 |  | 0.18 | 0.23 |  | 0.10 | NA |
| TGW | 0.27 | 0.34 |  | 0.11 | 0.17 |  | 0.01 | 0.06 |  | 0.23 | 0.22 |
|  |  |  |  |  |  |  |  |  |  |  |  |
| FT , flowering time; GY, grain yield; PH, plant height; Awns, presence/absence; Wkill, winter kill; MAT, maturity; Gpt, grain protein content;Ears, ears per metre squared; ; LR, lodging resistance; ; GSW, grain specific weight; ; TN, tiller number; TGW, thousand grain weight. | | | | | | | | | | | |

| **Supplementary Table S5.** Predictive correlations based on training populations assembled by country and comparing GBS and DArT markers.  (GBR =70, DEU = 82 and FRA = 192). | | | | | | |
| --- | --- | --- | --- | --- | --- | --- |
|  | GBS | | | DArT | | |
|  | FRA | | | FRA | | |
| Traits | GBR | DEU | GBR+DEU | GBR | DEU | GBR+DEU |
|  |  |  |  |  |  |  |
| FT | 0.30 | 0.17 | 0.25 | 0.22 | 0.24 | 0.24 |
| PH | 0.50 | 0.50 | 0.58 | 0.43 | 0.50 | 0.53 |
| GY | 0.48 | 0.68 | 0.61 | 0.49 | 0.61 | 0.54 |
| Awns | 0.55 | 0.04 | 0.33 | 0.59 | NA | 0.33 |
| Wkill | 0.16 | 0.17 | 0.41 | 0.13 | 0.35 | 0.53 |
|  |  |  |  |  |  |  |
| MAT | 0.11 | 0.34 | 0.35 | 0.04 | 0.25 | 0.28 |
| Gpt | 0.47 | 0.67 | 0.65 | 0.49 | 0.53 | 0.53 |
|  |  |  |  |  |  |  |
|  |  |  |  |  |  |  |
| Ears | 0.27 | 0.10 | 0.20 | 0.26 | 0.27 | 0.23 |
|  |  |  |  |  |  |  |
|  |  |  |  |  |  |  |
| LR | 0.46 | 0.29 | 0.41 | 0.32 | 0.28 | 0.31 |
| GSW | 0.15 | 0.24 | 0.31 | 0.18 | 0.26 | 0.35 |
| TN | 0.27 | 0.19 | 0.17 | 0.17 | 0.26 | 0.19 |
| TGW | 0.24 | 0.06 | 0.18 | 0.35 | 0.09 | 0.25 |
|  |  | | |  | | |
|  | GBS | | | DArT | | |
|  | DEU | | | DEU | | |
| Traits | GBR | FRA | GBR+FRA | GBR | FRA | GBR+FRA |
| FT | NA | 0.08 | 0.04 | 0.09 | 0.12 | 0.13 |
| PH | 0.62 | 0.36 | 0.43 | 0.39 | 0.38 | 0.39 |
| GY | 0.39 | 0.63 | 0.60 | 0.16 | 0.53 | 0.48 |
| Awns | NA | NA | NA | NA | NA | NA |
| Wkill | 0.01 | 0.15 | 0.10 | NA | 0.16 | 0.10 |
| MAT | 0.33 | 0.29 | 0.41 | 0.47 | 0.24 | 0.39 |
| Gpt | 0.36 | 0.48 | 0.49 | 0.34 | 0.38 | 0.40 |
| Ears | 0.02 | NA | NA | 0.10 | 0.11 | 0.10 |
| LR | 0.30 | 0.19 | 0.23 | 0.17 | 0.34 | 0.31 |
|  |  |  |  |  |  |  |
| GSW | 0.05 | 0.20 | 0.17 | NA | 0.16 | 0.13 |
| TN | 0.15 | 0.13 | 0.13 | 0.08 | 0.17 | 0.14 |
| TGW | 0.30 | 0.08 | 0.17 | 0.17 | 0.12 | 0.17 |
|  |  |  |  |  |  |  |
|  |  |  |  |  |  |  |
|  |  |  |  |  |  |  |
|  |  | | |  | | |
|  | GBS | | | DArT | | |
|  | GBR | | | GBR | | |
|  | FRA | DEU | FRA+DEU | FRA | DEU | FRA+DEU |
| FT | 0.29 | NA | 0.18 | 0.32 | 0.01 | 0.21 |
| PH | 0.44 | 0.59 | 0.51 | 0.45 | 0.52 | 0.48 |
| GY | 0.58 | 0.50 | 0.55 | 0.46 | 0.19 | 0.42 |
| Awns | 0.35 | NA | 0.20 | 0.41 | NA | 0.25 |
| Wkill | 0.17 | 0.03 | 0.10 | 0.09 | NA | 0.04 |
| MAT | 0.04 | 0.38 | 0.13 | 0.11 | 0.33 | 0.17 |
| Gpt | 0.48 | 0.53 | 0.42 | 0.39 | 0.45 | 0.40 |
| Ears | 0.14 | NA | 0.05 | 0.07 | 0.04 | 0.06 |
| LR | 0.45 | 0.25 | 0.39 | 0.37 | 0.17 | 0.32 |
| GSW | 0.24 | 0.15 | 0.24 | 0.21 | 0.05 | 0.18 |
|  |  |  |  |  |  |  |
| TN | 0.15 | 0.14 | 0.15 | 0.13 | 0.02 | 0.09 |
| TGW | 0.18 | 0.26 | 0.20 | 0.23 | 0.11 | 0.21 |
|  |  |  |  |  |  |  |
|  |  |  |  |  |  |  |
|  |  |  |  |  |  |  |

| Supplementary Table 6: List of DArT and GBS markers correlated. DArT markers are as identified in Bentley et al. 2014 | | | |
| --- | --- | --- | --- |
| GBS/DArT marker acronym | Marker name | Chromosome | Trait/s |
| G1B_434.FT | 1B_43412 | 1B | FT |
| G1B_408.FT | 1B_40806 | 1B | FT |
| G2D_314.FT/PH | 2D_31468 | 2D | FT/PH |
| G2D_420.FT/PH | 2D_42097 | 2D | FT/PH |
| G4B_213.PH | 4B_21378 | 4B | PH |
| G5A_226.PH | 5A_22657 | 5A | PH |
| G6A_223.PH | 6A_22347 | 6A | PH |
| G6A_422.PH | 6A_42275 | 6A | PH |
| G6A_101.PH | 6A_10143 | 6A | PH |
| G6A_112.Gpt | 6A_11268 | 6A | Gpt |
| G6A_151.Gpt | 6A_15149 | 6A | Gpt |
| G6A_448.Gpt | 6A_44801 | 6A | Gpt |
| G6A_882.GY | 6A_88260 | 6A | GY |
| G7B_631.GY | 7B_63134 | 7B | GY |
| D1B_wPt191.FT | wPt_1912 | 1B | FT |
| D2D_BW2525.FT | BWS2525_AC | 2D | FT |
| D2D_wPt733.FT | wPt_733227 | 2D | FT |
| D2D_PpdD1.FT/PH | PpdD1_297 | 2D | FT/PH |
| D4B_RhtB1.PH | Rht1_267 | 4B | PH |
| D5A_BW2245.PH | BWS2245 | 5A | PH |
| D5A_BW5515.PH | BWS5515 | 5A | PH |
| D6A_BW2952_PH | BWS2952 | 6A | PH |
| D6A_wPt.732.PH | wPt_732355 | 6A | PH |
| D6A_BW3158.GY/Gpt | BWS3158 | 6A | GY/Gpt |
| D7B_W2641.GY | BWS2641 | 7B | GY |

Supplementary Figures

**Figure S1**. Distribution of SNPs by minor allele frequencies within the final GBS dataset. The minimum MAF = 0.05


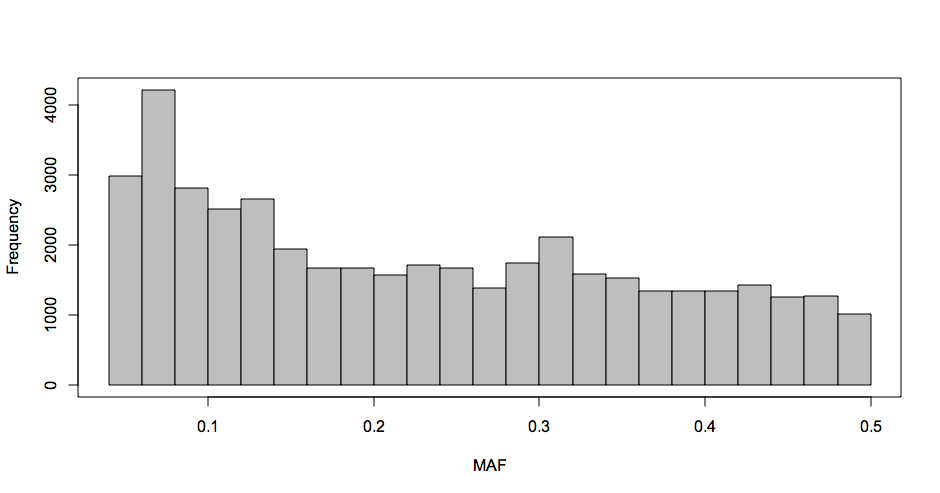


**Figure S2.** LD decay plots of r² over physical distance in mega base pairs (Mbp) on chromosomes 2A to 7D. The solid black line is the LD decay curve fitted by non-linear least squares. The black dotted line touches the curve at a point corresponding to the distance where LD decayed to r², 0.2.


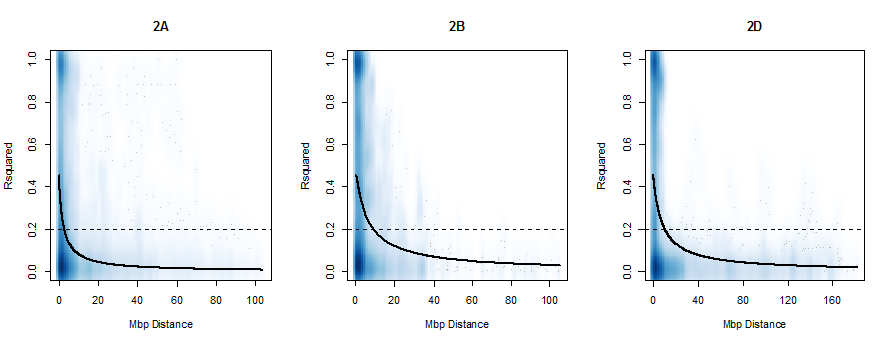


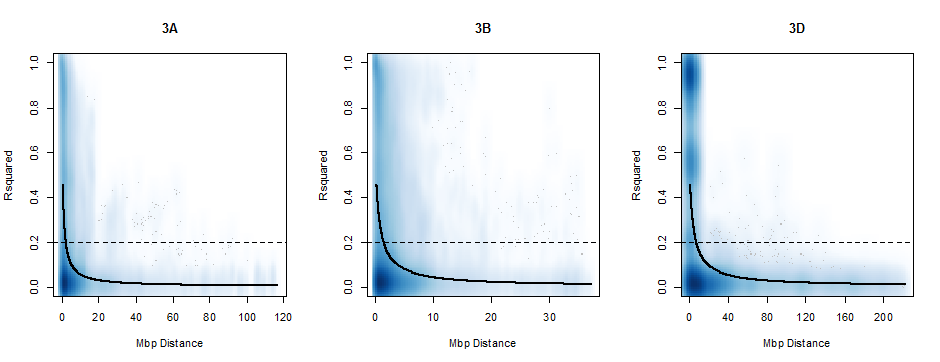


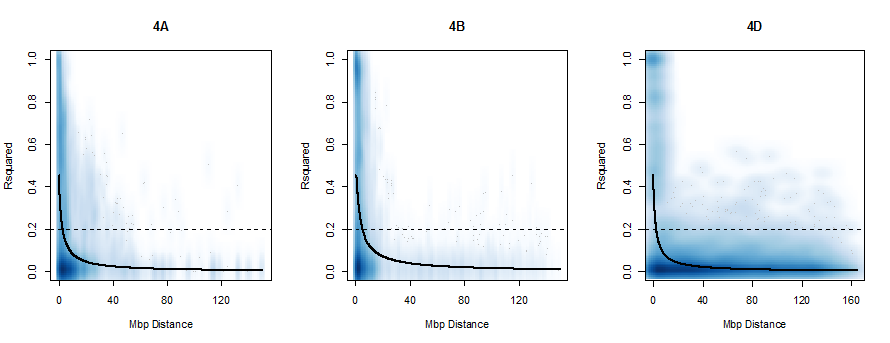


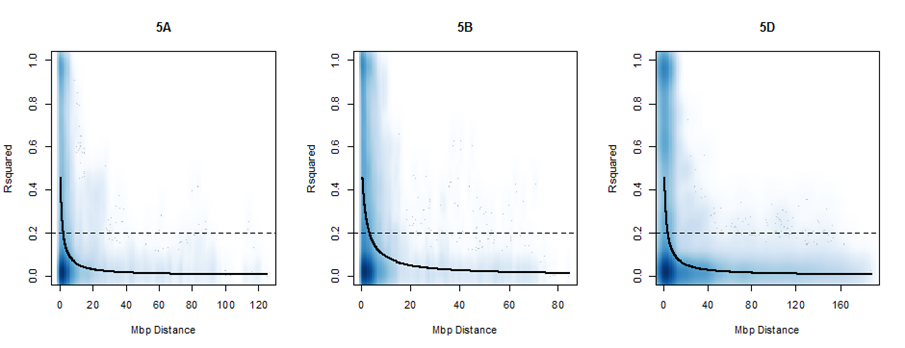


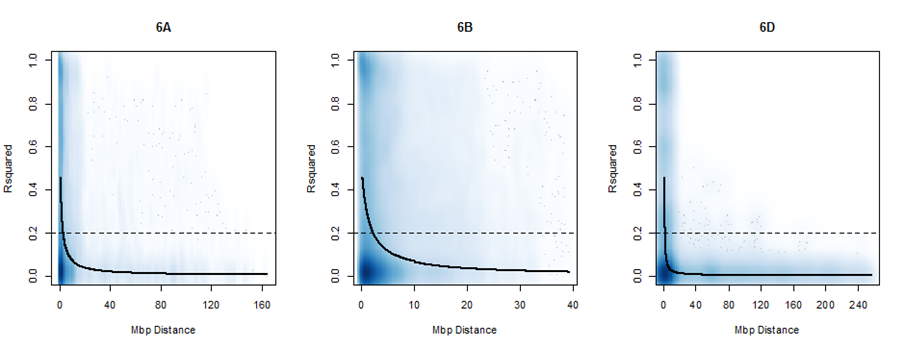


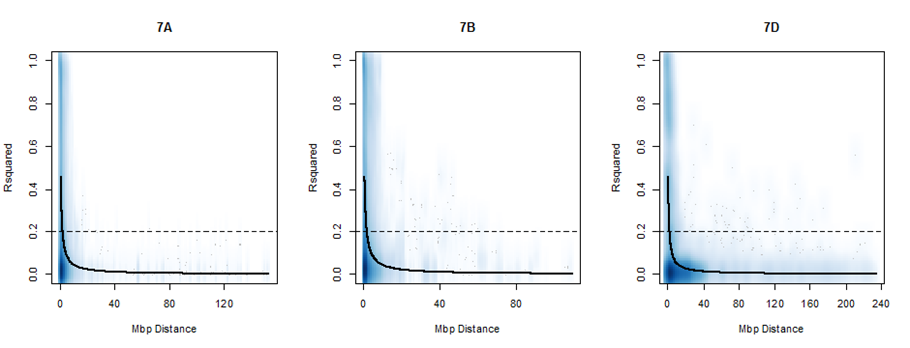


**Figure S3.** Graphical representation of the actual number of populations (K) as determined using STRUCTURE and the 7869 SNP dataset. ΔK (deltaK) calculated according to Evanno et al. (2005). The optimal K = 4.


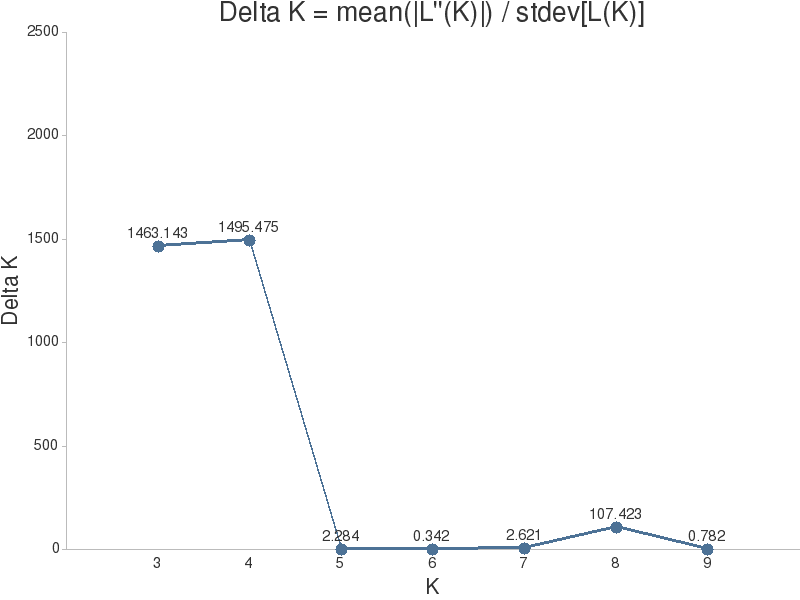


**Figure S4.** GWAS Manhattan plots for awn (presence/absence), winter kill, lodging resistance, grain protein, maturity and grain yield. The green line represents the experiment-wide Bonferroni threshold (p-value = 0.01) and the black line represents the FDR threshold p-value (0.05).


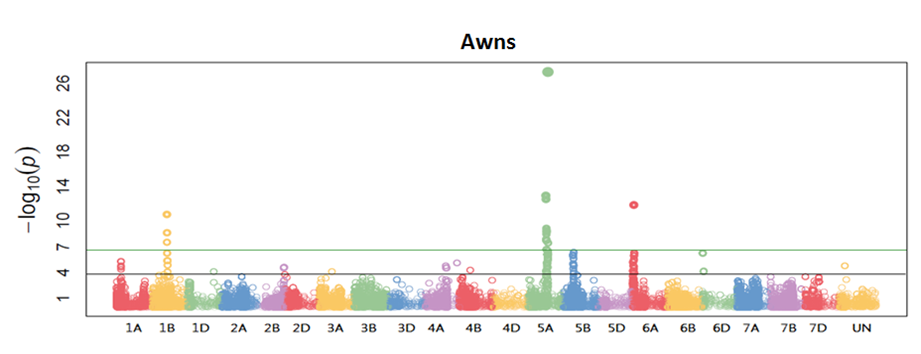


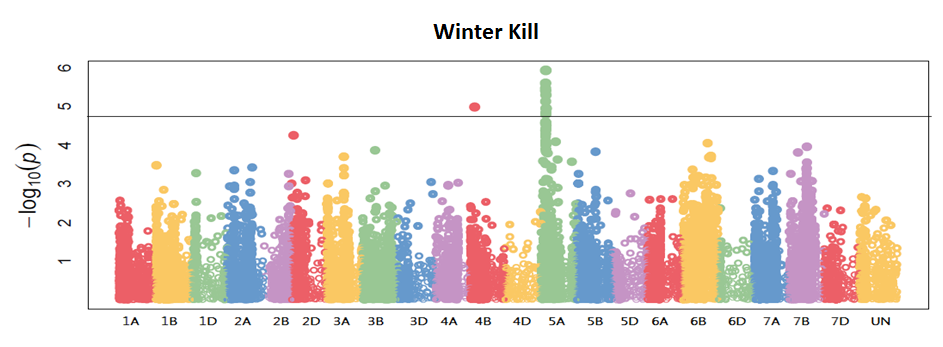


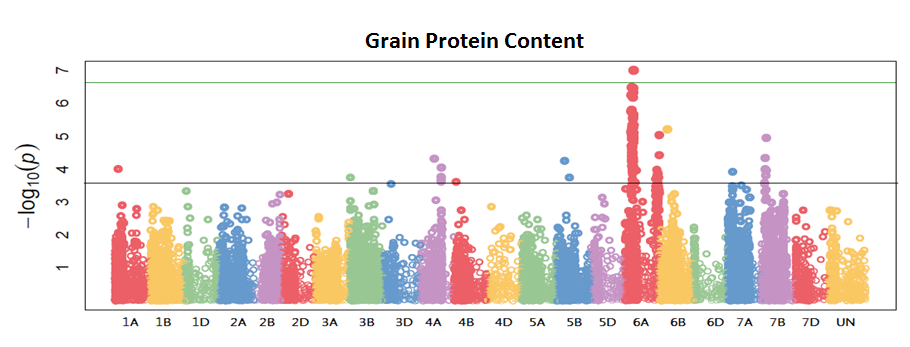


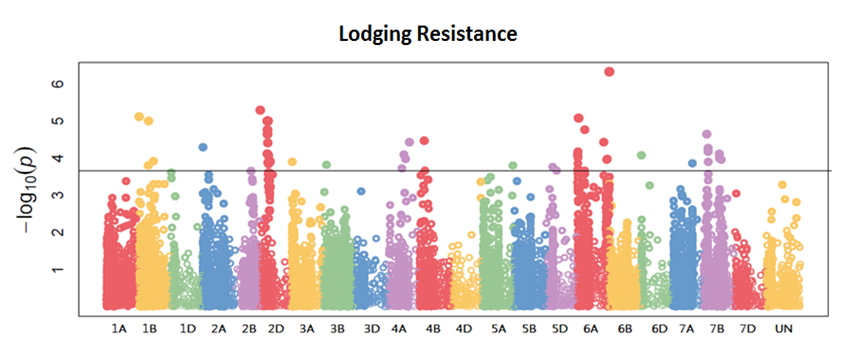


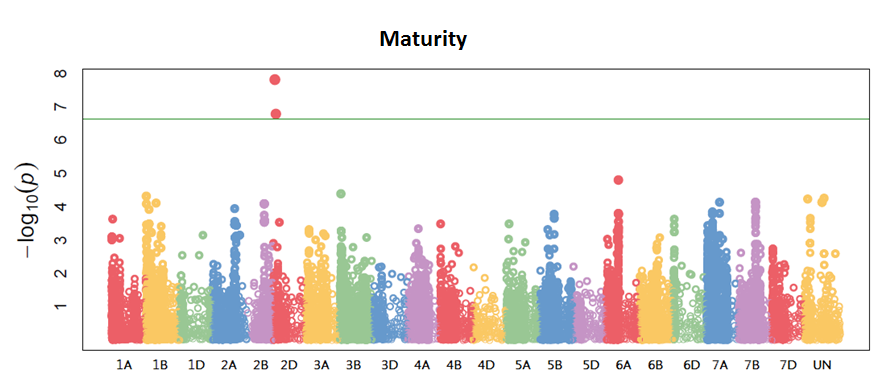


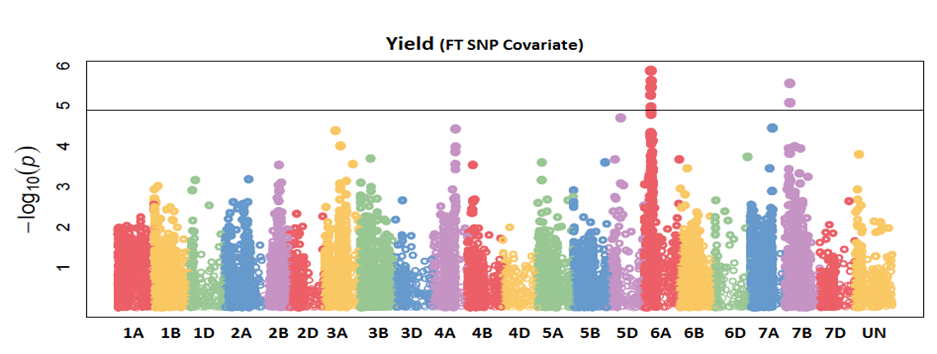


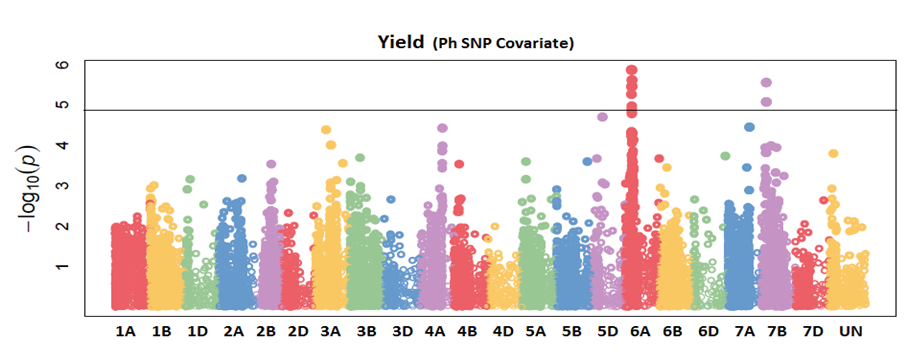

Supplement: Supplementary file 1 [file Table_1.docx]
